# Restoring Parkin function: an AAV gene therapy approach for early-onset Parkinson’s disease

**DOI:** 10.3389/fnmol.2026.1939065

**Published:** 2026-10-21

**Authors:** Sambuddha Basu, Tyler G. Demarest, Nicholas J. Gattone, Alexandra J. Gilsrud, Brittany Wicks, Apeksha Khatiwada, Mohammad Nayal, Renee Gentzel, Daniel M. Cohen, Eric W. Kostuk, Derek P. Narendra, Pedro Gonzalez-Alegre, Maria-Grazia Biferi, Elizabeth A. Ramsburg

**Affiliations:** 1 Spark Therapeutics, Philadelphia, PA, United States; 2 Roche Innovation Center (RICP), Philadelphia, PA, United States; 3 National Institutes of Health (NIH), National Institute of Neurological Disorders and Stroke (NINDS), Bethesda, MD, United States

**Keywords:** AAV gene therapy, gene replacement, mitophagy, Parkin (PARK2), Parkinson’s disease

## Abstract

Biallelic loss-of-function mutations in *PRKN* gene (encoding Parkin protein) cause early-onset Parkinson’s disease (EOPD). Parkin is a crucial component of the PINK1-Parkin pathway, which marks damaged mitochondria for degradation via mitophagy. Without functional Parkin, damaged mitochondria accumulate, causing oxidative stress and neurodegeneration. We investigated Parkin gene replacement via AAV gene therapy as a potential treatment for Parkin-dependent EOPD. We validated phosphorylated ubiquitin Ser65 (pUb^Ser65^) as an indicator of PINK1-Parkin pathway initiation/activation. We evaluated AAV-mediated *PRKN* replacement (AAV-Parkin) in a Parkin knockout neuroblastoma cell (SH-SY5Y cells) and feasibility of delivery in mouse and rat models. Our research showed pUb^Ser65^ signal was reduced in Parkin-KO SH-SY5Y cells when compared to wild-type cells after mitochondrial stress, indicating deficiency in initiation of mitophagy. AAV-mediated human *PRKN* gene replacement restored these pUb^Ser65^ levels in knockout cells. We saw restoration in patient-derived fibroblasts following AAV-Parkin overexpression. We developed a translatable gene therapy approach using rodents. We demonstrated the feasibility of delivering AAV-Parkin directly into the substantia nigra (SN) of wild-type rats. Using an AAV1 capsid with Ef1a promoter, we achieved dose-dependent Parkin expression and identified a well-tolerated dose. We also evaluated multiple promoters in a proprietary Spark100 capsid (also referred as Spk100), finding Ef1a and Synapsin1 (Syn1) were most effective for transducing relevant cell populations, including dopaminergic neurons, in the SN of mice without causing adverse effects. These findings established a short-term well-tolerated vector dose and an optimal capsid-promoter combination. Our results establish foundational feasibility and short-term tolerability, supporting the potential of AAV-Parkin gene therapy as a promising candidate for further preclinical development for Parkin-deficient EOPD.

## Graphical abstract

**Figure:**
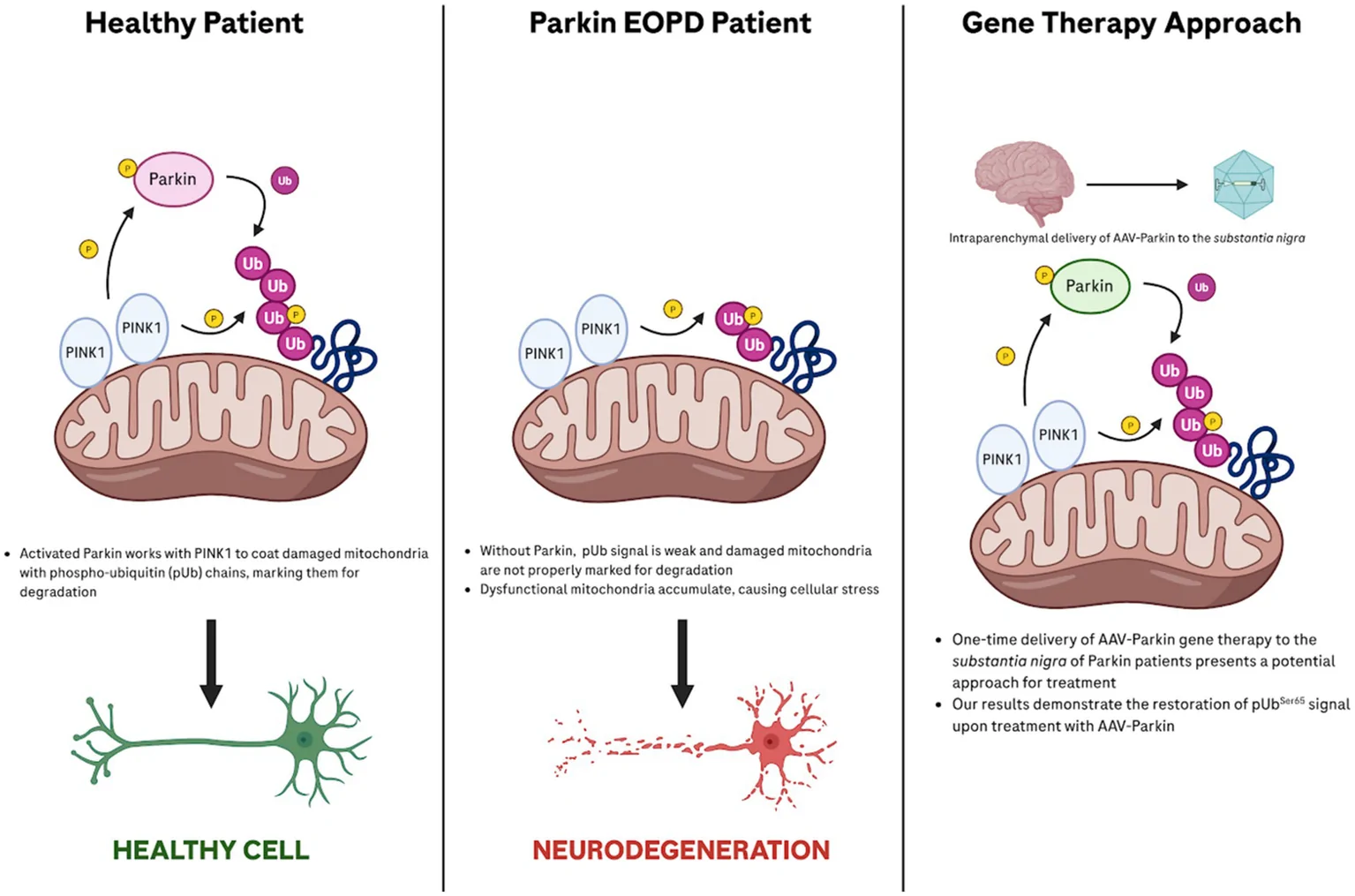

## 1 Introduction

Parkinson’s disease (PD) is a progressive neurodegenerative disorder characterized by motor symptoms including bradykinesia, rigidity, tremor, and postural instability caused by degeneration of dopaminergic neurons in the substantia nigra pars compacta (SN/SNpc) (Jankovic, 2008). While the majority of PD cases are classified as idiopathic and late-onset, a subset of patients exhibit early-onset Parkinson’s disease (EOPD), defined as the onset of symptoms before the age of 50 (Schrag and Schott, 2006). EOPD is clinically and genetically distinct from late-onset PD, with a higher likelihood of genetic contributions playing a central role in its pathogenesis (Klein and Schlossmacher, 2006).

A commonly mutated gene in EOPD is *PRKN*, which in some populations is found in approximately 2.6–14.9% of EOPD cases (Lücking et al., 2000; Koros et al., 2017; Menon et al., 2024). Epidemiological studies provide insight into the prevalence of *PRKN* mutations. Two recent studies estimated the genetic prevalence of PRKN-PD to be approximately 13 per 100,000 (Diogo et al., 2024; Zhu et al., 2022). The *PRKN* gene, which encodes E3 ubiquitin ligase Parkin, plays a crucial role in maintaining cellular homeostasis through the regulation of protein degradation and mitochondrial function (Lücking et al., 2000; Narendra et al., 2008; Oshima et al., 2021). Loss-of-function mutations in the *PRKN* gene lead to impaired mitophagy, an essential process for the removal of damaged mitochondria, which contributes to the accumulation of defective proteins and cellular stress, ultimately resulting in neurodegeneration and EOPD (Kitada et al., 1998). The PINK1-Parkin pathway is a crucial process for clearing damaged mitochondria through mitophagy (Bernardo et al., 2024). When mitochondria are stressed, PINK1 accumulates on their surface and phosphorylates ubiquitin at residue Serine 65 (pUb^Ser65^), a key signal for the initiation of mitophagy. Binding phosphorylated ubiquitin is the first event required for Parkin activation. PINK1 then directly phosphorylates Parkin itself, providing the second signal required for activation of Parkin’s E3 ligase activity. Parkin, once activated, ubiquitinates proteins on the mitochondrial surface, providing an additional substrate for PINK1. This combined action greatly amplifies the damage signal, recruiting more Parkin to the damaged mitochondria and initiating the full mitophagy process (Kazlauskaite et al., 2014; Koyano et al., 2014; Kane et al., 2024). The accumulation of pUb^Ser65^ requires PINK1 but is enhanced by endogenous Parkin.

Disease-causing mutations in *PRKN* range from single base pair substitutions, small deletions, and splice site mutations to deletions and duplications spanning many nucleotides (Menon et al., 2024). *PRKN* mutations lead to a loss of Parkin function. For missense mutations, loss of Parkin function may be due to decreased catalytic activity and/or destabilization of Parkin leading to insolubility or rapid proteasomal degradation of mutant Parkin. Gene therapy using adeno-associated virus (AAV) vectors offers a promising strategy for treating *PRKN*-associated EOPD by delivering functional *PRKN* gene (and Parkin protein) to the SN. AAV vectors are particularly well-suited for gene therapy due to their safety, ability to transduce both neuronal and non-neuronal cells, and long-term expression in the target tissue (Mandel and Burger, 2004; Hocquemiller et al., 2016; Zhu et al., 2021; Naso et al., 2017). Direct delivery of *PRKN* gene (and Parkin protein) to the SN can potentially restore its function in dopaminergic neurons where endogenous Parkin function is lost, enhancing mitochondrial quality control and promoting the clearance of damaged mitochondria through mitophagy (Exner et al., 2012). This approach could mitigate neurodegeneration of dopaminergic neurons and the development of PD symptoms. Gene-therapy approaches delivering AAV vector payload directly to SN and dopaminergic neurons have not only been well-established in clinics but also deemed safe and a reasonable translatable approach (Murlidharan et al., 2014; Pearson et al., 2021).

In this manuscript, we evaluate AAV gene therapy for *PRKN*-associated EOPD. Our findings highlight increased pUb^Ser65^ as an indicator of PINK1-Parkin pathway activation when mitochondria are stressed. We further show that AAV-Parkin gene therapy can restore this activation in Parkin null cells or Parkinson’s patient-derived cells. Building on our in-vitro findings, we next optimized promoters to ensure the efficient expression of functional Parkin in relevant cell types, including dopaminergic neurons, in the SN of wild-type rodents. Our foundational hypothesis is that restoring at least 50% of endogenous functional Parkin protein expression in dopaminergic neurons will be therapeutically beneficial. This is supported by human genetics, which has established that Parkinson’s disease (PD) only develops in individuals with biallelic loss of function in the *PRKN* gene (Zhu et al., 2022). This clinical observation provides a rationale for our gene therapy approach. We demonstrate *in vivo* feasibility and dose-finding studies to achieve targeted 50% endogenous Parkin expression in the SN of rats. Our work demonstrates *in vitro* proof of technical principle and outlines a clear path for developing AAV-Parkin gene therapy as a potential treatment for *PRKN*-deficient EOPD.

## 2 Materials and methods

Detailed descriptions of all materials and methods can be found in Supplementary material. These include assessment of WT and Parkin knock out SH-SY5Y cells, patient derived fibroblasts from healthy controls and Parkin-PD patients for initiation of mitophagy signal, and tolerability assessment of AAV-Parkin gene therapy and promoter optimization. A detailed method for the vector production is included in the supplementary material and methods (Alam et al., 2025).

## 3 Results

### 3.1 AAV-Parkin restores pUb^Ser65^ signal and mitophagy initiation following mitochondrial stress in Parkin-deficient cells

To determine a cellular phenotype consistent with Parkin deficiency, we evaluated a commercially available Parkin knockout (KO) SH-SY5Y neuroblastoma cell line. Initial experiments revealed no differences in mitophagy and ubiquitin proteasome system endpoints under basal conditions between WT and Parkin KO cells treated with DMSO (Figure 1A). We then evaluated the addition of mitochondrial stress to mimic the age-related accumulation of damaged mitochondria that contributes to dopaminergic neuron dysfunction as observed in *PRKN*-EOPD patients. The loss of mitochondrial membrane potential limits cellular energy production in the form of ATP and initiates the removal of uncoupled mitochondria through both Parkin-dependent and Parkin-independent mitophagy pathways. Therefore, we evaluated the pharmacological proton ionophore carbonyl cyanide 4-(trifluoromethoxy) phenylhydrazone (FCCP) to collapse mitochondrial membrane potential and mimic age-related mitochondrial impairments, acting as a stimulus to initiate PINK1-dependent, Parkin-amplified mitophagy. FCCP was specifically selected for the SH-SY5Y cell line following empirical testing, as it provided the strongest and most consistently measurable pUb^Ser65^ response window compared to other uncouplers during our assay optimization. We identified a significant Parkin-mediated increase in pUb^Ser65^ in WT cells only, that was not seen in the Parkin KO neuroblastoma cells. This is consistent with literature demonstrating pUb^Ser65^ production by PINK1 can be amplified by Parkin (Exner et al., 2012).

**Figure 1:**
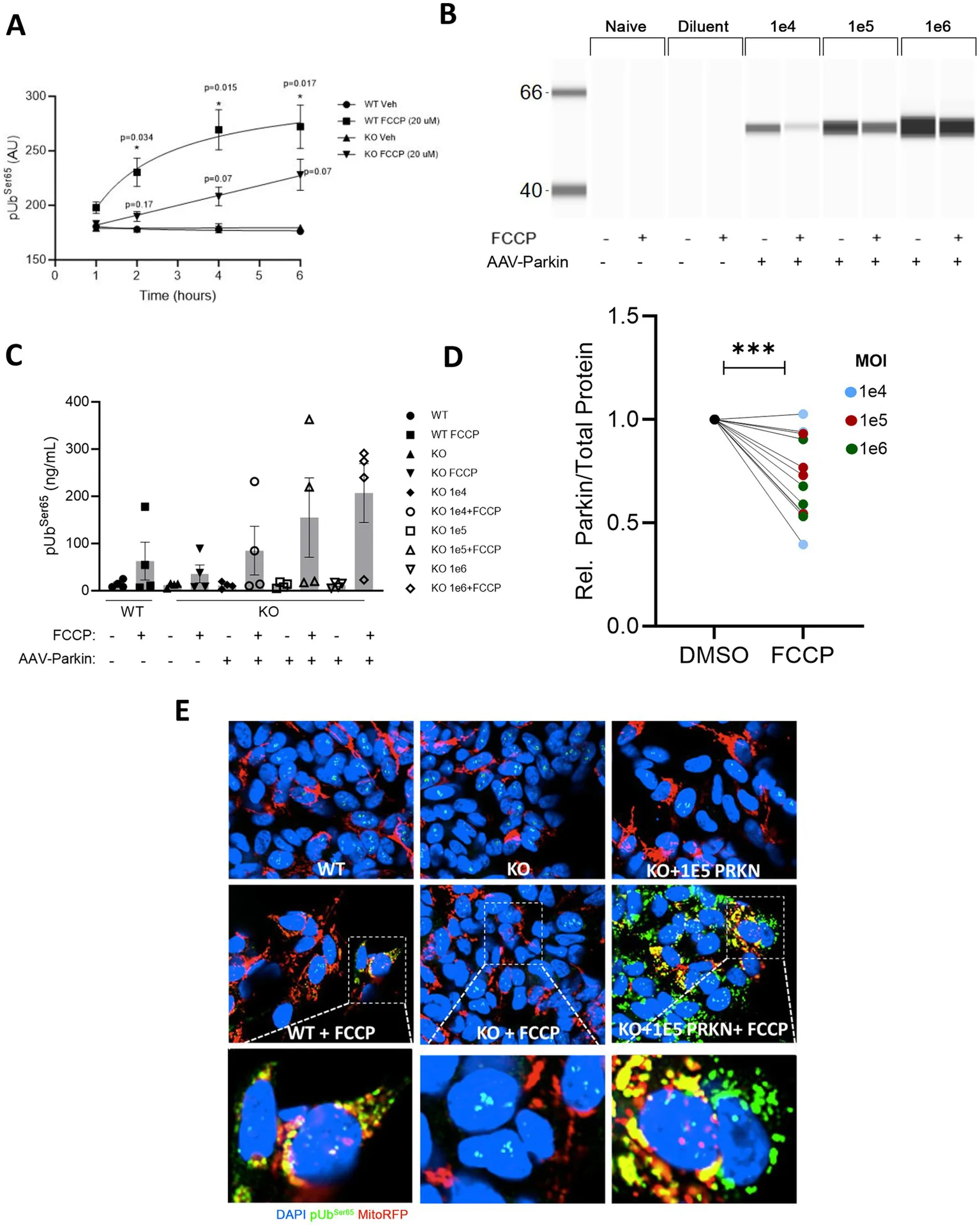
AAV-Parkin (human) rescues pUb^Ser65^ deficiency following mitochondrial stress in Parkin knockout (KO) SH-SY5Y neuroblastoma cells. **(A)** Time-course identification of pUb^Ser65^ deficiency in Parkin KO cell lysates following vehicle or FCCP (20 μM) by dot blot. **(B)** AAV1-Ef1a-Parkin (human) transduction of Parkin KO cells reveals MOI-dependent quantitative expression of Parkin and **(C)** corresponding quantitative pUb^Ser65^ levels assessed by ELISA. *p < 0.05, 2-way ANOVA vs. vehicle treatment, RM-2-ay ANOVA with Tukey’s *post hoc* test. **(D)** Parkin protein is reduced following FCCP treatment reflecting Parkin degradation following mitochondrial stress in KO cells overexpressing Parkin by AAV-Parkin. ***p = 0.0005, Student’s *t*-test DMSO vs. FCCP in KO cells expressing Parkin by AAV-Parkin. *n* = 12 independent lysates. **(E)** pUb^Ser65^ co-localizes with mitochondria-RFP following FCCP treatment.

An initial time-course evaluation of pUb^Ser65^ by dot-blot analysis demonstrated induction of pUb^Ser65^ in WT cells (starting at 2 h and peaking at 4 h) when challenged with 20 μM FCCP, compared to DMSO treated WT cells, indicating a normal initiation of the PINK1-Parkin signaling cascade (Figure 1A, WT vs. WT + FCCP, *p* < 0.05 at 2 h, 6 h and 8 h, Supplementary Figure 1A). However, in Parkin KO cells, this showed a non-significant change in pUb^Ser65^ signal following FCCP treatment at all time-points compared to control Parkin KO cells with DMSO (Figure 1A, KO vs. KO + FCCP, *p* > 0.05 for all time points, Supplementary Figure 1A). This result was confirmed qualitatively through representative pUb^Ser65^ immunofluorescence staining (Supplementary Figure 1B, panel 1 and 2: top and bottom). After endpoint identification of PINK1-dependent, Parkin-amplified pUb^Ser65^ production, we interrogated whether deficiency in pUb^Ser65^ amplification could be restored by introducing a functional copy of the *PRKN* gene using gene therapy.

WT and Parkin KO cells were transduced with 3 multiplicity of infection (MOI) of AAV1-Parkin (human) driven by the ubiquitous promoter Ef1a. Following 72 h of transduction, cells were treated with FCCP or DMSO (vehicle control) and harvested for Parkin protein expression and pUb^Ser65^ determination by ELISA. We observed an MOI-dependent increase in Parkin protein expression (Figure 1B), and similar dose-dependent trends for pUb^Ser65^ upregulation following FCCP KO 1e6 versus KO 1e6 + FCCP (*p* < 0.05), but not vehicle treatment, indicating AAV-Parkin was able to upregulate pUb^Ser65^ (Figure 1C). This amplification of pUb^Ser65^ signal was visually confirmed through pUb^Ser65^ immunofluorescence staining in Parkin KO cells treated with optimal MOI of AAV1-Ef1a-Parkin (human) and challenged with FCCP (Supplementary Figure 1B, panel 3: top and bottom). We then investigated whether downstream clearance of AAV-Parkin, an indirect marker of mitochondrial cargo processing, occurred following cellular stress. Notably, we observed a statistically significant reduction of Parkin protein in the cells treated with FCCP compared to the DMSO control (Figure 1D, *p* = 0.0005), indicating the gene therapy delivered Parkin was likely binding to dysfunctional mitochondria and being degraded alongside mitochondrial cargo via mitophagy. To further investigate the mitochondrial localization of pUb^Ser65^, we performed co-staining experiments with mitochondrial-tagged red fluorescence protein (mito-RFP) and pUb^Ser65^ in Parkin KO cells following AAV-Parkin transduction treated with DMSO versus FCCP. We observed absence of pUb^Ser65^ staining in all cells under basal condition, which is in line with the ELISA results. Importantly, we observed mitochondrial co-localization of pUb^Ser65^ after AAV-delivered Parkin protein following FCCP treatment (colocalization of mito-RFP and green signal of pUb^Ser65^, Figure 1E). Together, these experiments demonstrate pUb^Ser65^ amplification is deficient in Parkin KO neuroblastoma cells following mitochondrial damage, but can be restored following AAV-Parkin treatment.

### 3.2 AAV-Parkin restores pUb^Ser65^ amplification signal in patient-derived fibroblasts from EOPD (PRKN-PD) following mitochondrial stress

While the initial results suggest a promising approach for the restoration of Parkin function in Parkin KO SH-SY5Y cells, patient-relevant *PRKN* gene mutations contributing to EOPD are diverse. This genetic complexity may not be fully recapitulated in a KO cell system. Therefore, we evaluated patient-derived fibroblasts containing distinct *PRKN* mutations (*n* = 8-9), or fibroblasts from healthy control non-Parkin mutation carriers (*n* = 4) (Supplementary file 2). All patient lines contained biallelic mutations in either a recessive or compound heterozygous state. To study how Parkin affects initiation of mitophagy, we first had to find an efficient way to transduce patient-derived fibroblasts. We compared different AAV (adeno-associated virus) capsids and found that AAV2 was the most effective. This vector, which initially used a CAG promoter to express a GFP (green fluorescent protein) transgene, proved successful (Supplementary Figure 3A). With this optimized method, we created a standardized procedure to deliver the human *PRKN* gene into the fibroblasts using the same AAV2-CAG vector. For the next round of experiments, we treated these cells with valinomycin, a mitochondrial uncoupler, to observe how the newly introduced Parkin protein would respond to mitochondrial stress, as shown in the schematic image (Figure 2A). Similar to our optimization in SH-SY5Y cells, valinomycin was chosen for the primary fibroblasts because prior optimizations based on established primary fibroblast protocols (Rakovic et al., 2011) revealed it yielded a more robust and reliable dynamic range for pUb^Ser65^ activation compared to other standard uncouplers like CCCP.

**Figure 2:**
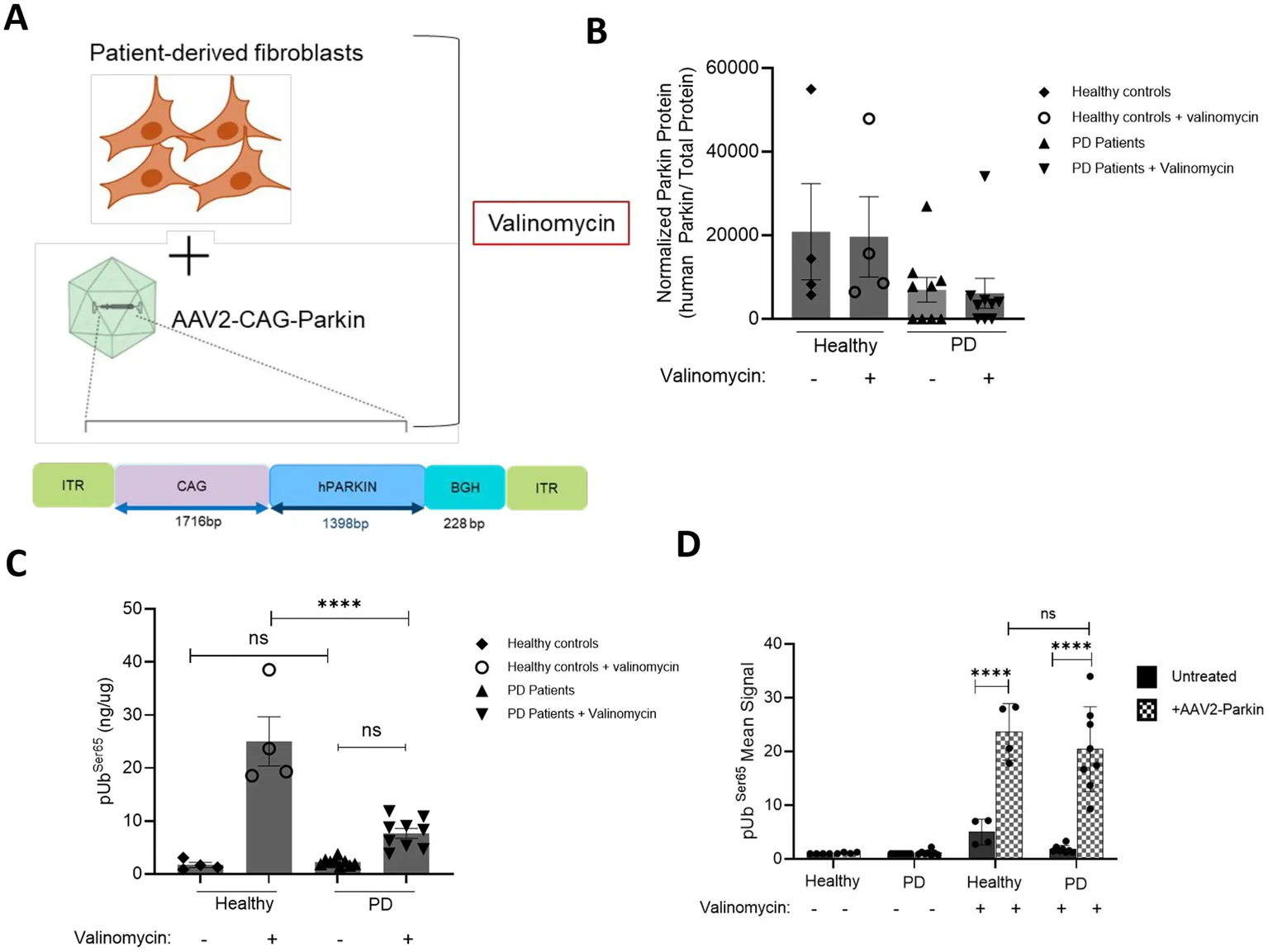
AAV-Parkin restores pUb^Ser65^ deficiency following mitochondrial stress in PD patient-derived fibroblast lines. **(A)** Graphical representation showing fibroblast lines from PD patients and controls from non-PD first-degree relatives were treated with AAV2-CAG-Parkin (human) and stressed with valinomycin. **(B)** Quantified Parkin assessment by capillary electrophoresis shows no change between PD patients and controls from non-PD, and also after stress with valinomycin. **(C)** pUb^Ser65^ by ELISA showing deficit of pUb^Ser65^ in fibroblasts from PD patients when compared to controls from non-PD with valinomycin treatment. ****p < 0.0001, ordinary one-way ANOVA Tukey’s multiple comparisons test. No statistically meaningful difference was observed between PD patients with and without valinomycin treatment (*p* = 0.0665). **(D)** Quantified mean signal from pUb^Ser65^ ICC staining demonstrating restoration of pUb^Ser65^ signal comparable to WT levels when cells are treated with AAV2-CAG-Parkin (human) and stressed with valinomycin. ****p < 0.0001 way ANOVA Tukey’s multiple comparisons test. Selected statistical comparisons are shown on the graph.

Basal levels of Parkin protein expression were similar with and without valinomycin in Controls and PD patient lines. (Figure 2B). However, a significant reduction in pUb^Ser65^ signal was observed in Parkin mutation carriers compared to healthy controls when challenged with valinomycin, when measured by both ELISA and immunocytochemistry (Figure 2C, *p* < 0.0001, Supplementary Figure 3B). We did not observe any difference in the basal level of pUb^Ser65^ between Controls and PD-patient cells. Furthermore, there was no statistically significant difference in pUb^Ser65^ levels in PD patient fibroblasts with and without valinomycin treatment (*p* = 0.0665). Following treatment with valinomycin, fibroblasts from PRKN-PD patients showed decreased production of pUb^Ser65^. The persistence of a residual pUb^Ser65^ signal in these patient fibroblasts (Figure 2) underscores that while PINK1 initiates pUb^Ser65^ generation, the robust amplification of this signal is absent without functional Parkin.

For the next set of experiments, we aimed at rescuing the pUb^Ser65^ production defect in *PRKN*-PD patients by transducing the fibroblasts with AAV2-CAG-Parkin (human, MOI: 1e4 particles/condition) (Figure 2D). We demonstrated by immunocytochemistry that replacing functional Parkin with AAV2-CAG-Parkin (human) rescued deficient pUb^Ser65^ amplification following valinomycin treatment in all *PRKN*-PD patient lines irrespective of the Parkin mutations. pUb^Ser65^ in *PRKN*-PD patient cells after rescue was comparable to the upregulation of pUb^Ser65^ signal observed in healthy control lines (Figure 2D, Supplementary Figures 3B,C, *p* < 0.0001). Individual data points from each fibroblast line post transduction with AAV2-CAG-Parkin (human) are shown in Supplementary Figure 3C. Together, these results demonstrate AAV-Parkin gene therapy restores Parkin activity in Parkin-mutant cells, irrespective of mutation.

### 3.3 Intraparenchymal substantia nigra (SN) injection of AAV-Parkin in rats is feasible and well tolerated

We next investigated the feasibility and tolerability of delivering human Parkin via AAV-gene therapy by stereotaxic intraparenchymal (IPa) injection in the SN. To evaluate this, we administered 3 doses (3e8, 3e9 and 3e10 vg/SN) of AAV1-Ef1a-Parkin (human) to 2-month-old rats. The animals’ body weights remained comparable after treatment (Figure 3A). We observed a dose-dependent increase in immunostaining of human Parkin protein (red), which co-localized with the dopaminergic neuron marker tyrosine hydroxylase (TH in green; Figure 3B), indicating successful delivery to SN. Using capillary electrophoresis western blotting, we quantified Parkin protein compared to naïve and diluent injected rat SN samples. Because our assay parameters selectively captured the overexpressed human transgene—as evidenced by the complete absence of signal in the naïve and diluent-treated control tissues—we ensured our measurements exclusively reflected the AAV-delivered therapy, which showed increasing dose-dependent expression of human Parkin protein. (Figure 3C, *p* < 0.05, Supplementary Figure 4A). To further understand the amount of human Parkin protein delivered in relation to endogenous levels of rat Parkin expression, we utilized an antibody that detects both human and rat Parkin protein. We observed that the 3e8vg/SN dose of AAV-Parkin resulted in Parkin expression ranging from 35–260% (mean = 135.25%) compared to endogenous rat Parkin, which was slightly above the desired range of 50–100%. These data suggest that the 3e8 vg/SN dose approximates our targeted therapeutic range. However, we acknowledge the substantial variability observed (35–260%, *n* = 4), which is likely inherent to stereotaxic delivery into a small, deep brain structure like the SN, meaning this dose does not uniformly guarantee strict physiological replacement (Figure 3D, Supplementary Figures 4B,H).

**Figure 3:**
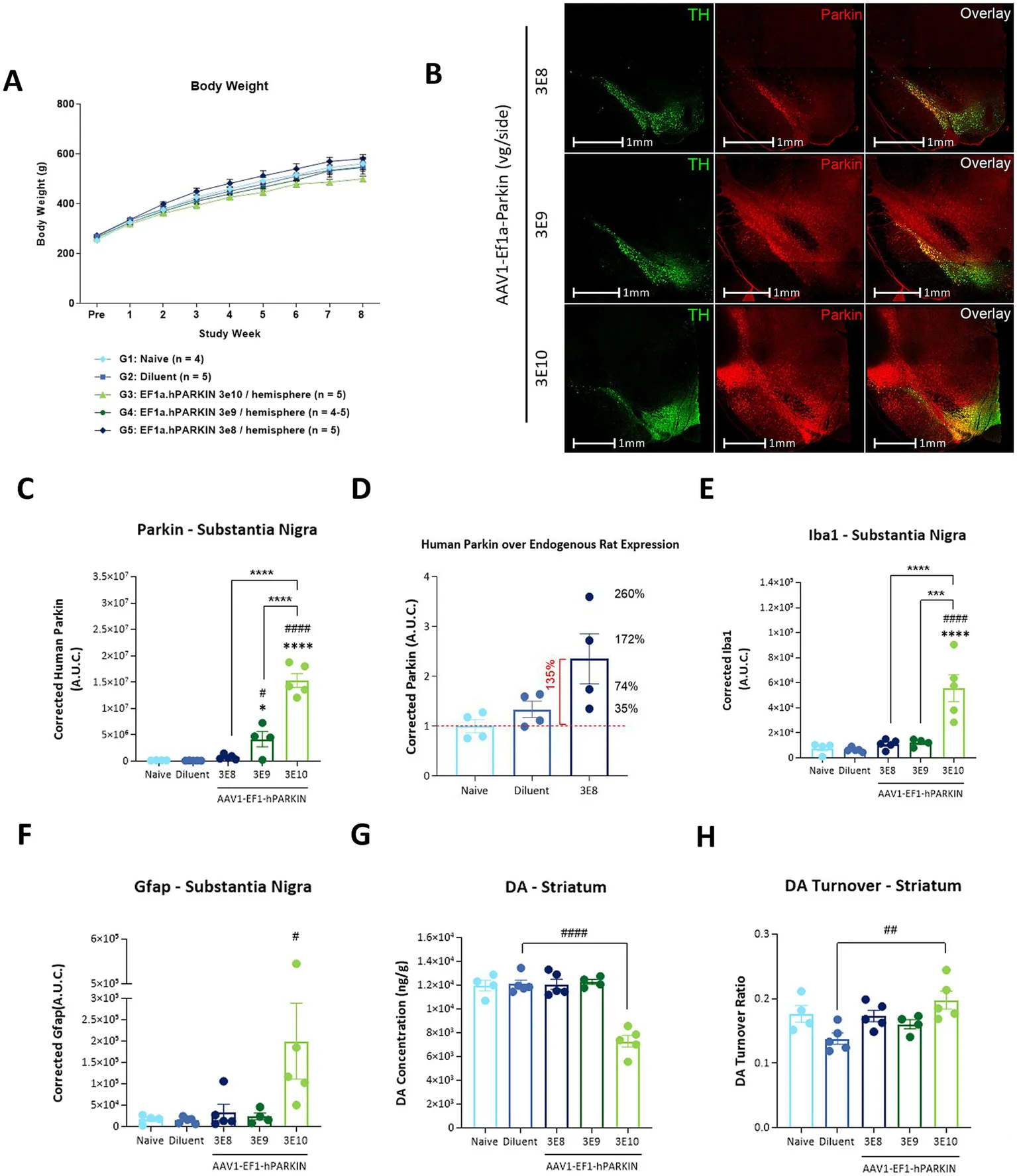
Dose-dependent Parkin (human) expression following stereotaxic delivery of AAV-Ef1a-Parkin (human) in rat substantia nigra pars compacta (SNpc). **(A)** Rats bilaterally injected in the SN using stereotaxic surgical procedures showed increased body weight over time. **(B,C)** Brain sections stained for TH (dopaminergic neuron marker, green) and human Parkin protein (red) shows dose-dependent increase of Parkin expression in SN and colocalization with dopaminergic neuron, which is also confirmed by capillary electrophoresis based biochemical assessment of human specific Parkin. 1-way ANOVA with Tukey’s *post hoc* analysis performed showing comparisons as Naïve vs. 3E9, *p = 0.0412; Naïve vs. 3E10, ****p < 0.0001 Diluent vs. 3E9, ^#^*p* = 0.0276; Diluent vs. 3E10, ^####^*p* < 0.0001; 3E8 vs. 3E10, ****p < 0.0001; 3E9 vs. 3E10, ****p < 0.0001; **(D)** Human Parkin and total Parkin (human and mouse) quantified by capillary electrophoresis in the 3e8 vg/SN cohort to determine therapeutic levels of Parkin delivered with a goal of reaching at least 50% Parkin expression. **(E,F)** To assess short-term tolerability, reactive markers for microglia (Iba1, **E**) and astrocytes (Gfap, **F**) were quantified by capillary electrophoresis. 1-way ANOVA with Tukey’s *post hoc* analysis performed showing comparisons of GFAP-Diluent vs. 3E10, ^#^*p* = 0.0471; IBA1-Naïve vs. 3E10, ****p < 0.0001; Diluent vs. 3E10, ^####^*p* < 0.0001; 3E8 vs. 3E10, ****p < 0.0001.; 3E9 vs. 3E10, ***p = 0.0003 **(G,H)** Striatal dopamine and dopamine turnover quantified by HPLC showed potential disruption of neurotransmitter synthesis at the highest dose of 3e10vg/SN only. 1-way ANOVA with Dunnett’s *post hoc* analysis performed showing comparisons of Dopamine: Diluent vs. 3E10, ^####^*p* < 0.0001 and Dopamine turnover: Diluent vs. 3E10, ^###^*p* = 0.0026.

To assess the tolerability of Parkin protein expression, we measured glial markers Gfap and Iba1 to screen for astrocyte and microglial activation, respectively. We observed significant upregulations of Iba1 (Figure 3E, Diluent vs. 3e10 vg, ^####^*p* < 0.0001, Supplementary Figure 4C) and Gfap (Figure 3F, ^#^*p* = 0.0471, Supplementary Figure 4D) at 3e10vg/SN only. Our study lacked a dose-matched empty vector control at the 3e10 vg/SN dose, meaning we cannot definitively separate the vector’s physical effect from that of Parkin protein overexpression in this specific cohort. Therefore, within the context of this rat study, we limit our interpretation to a direct association between high-dose AAV-Parkin treatment, subsequent gliosis, and dopaminergic dysfunction. However, we specifically address and resolve the mechanistic differentiation between vector-load and transgene-toxicity in our subsequent promoter-optimization study. As decreased striatal dopamine is a hallmark of nigrostriatal degeneration associated with PD, we quantified striatal dopamine and metabolites by HPLC. We observed no difference in dopamine or dopamine turnover ([DOPAC + HVA]/Dopamine) at 3e8 or 3e9 vg/SN compared to the Diluent group. However, consistent with the potentially adverse glial activation observed, we observed significant reduction in striatal dopamine (Figure 3G, *p* < 0.0001) and impaired dopamine turnover (Figure 3H, *p* = 0.0026) at the 3e10 vg/SN dose (Supplementary Figures 4E–G). Altogether, the results demonstrate that functional replacement of Parkin protein is feasible by direct SN IPa injection of AAV-Parkin starting at a dose of 3e8/SN and is well-tolerated short-term at doses below 3e10 vg/SN, giving a wide therapeutic window for *PRKN* gene replacement.

### 3.4 Ef1a and Syn1 promoters drive optimal Parkin expression in AAV vectors in the SN of wild-type mice

In the previous tolerability assessment, we utilized AAV1 serotype and a ubiquitous promoter (Ef1a) to drive the expression of the Parkin protein in dopaminergic neurons. We next sought to evaluate the expression profile of Parkin in relevant cell types, including dopaminergic neurons of the SN, by evaluating regulatory promoter sequences driving Parkin expression in a Spark proprietary capsid Spark100. Six AAV constructs expressing full length human Parkin driven by different promoters and a diluent control were delivered by intraparenchymal injection to the SN of wild-type mice at 1.0 × 10^10^ vg/SN. For comparability to prior experiments, we utilized the same AAV1-Ef1a-Parkin (human) construct injected in rats (Figure 3), and Spark100 vectors with 2 ubiquitous promoters (Ef1a or CAG), one neuron-specific promoter (human Synapsin; hSyn1), and 2 dopaminergic neuron-specific promoters (Tyrosine hydroxylase: TH—full length or mini) (Figure 4A) (Rolland et al., 2016). 8 weeks following AAV injection, mice were sacrificed and one hemibrain was collected for immunohistochemistry and the other for biochemical analysis. Parkin protein was assessed biochemically by capillary electrophoresis western blot. We observed the greatest Parkin expression in the SN with the ubiquitous CAG promoter, followed by Ef1a, TH-full length, and hSyn1, with a lack of expression by the TH-mini promoter, respectively, (Figure 4B, Supplementary Figures 5A,B,I). To further assess successful expression within dopaminergic neurons, we immunostained brain sections. These showed colocalization of human Parkin protein (red) with TH positive dopaminergic neurons (green), supporting the biochemical assessment of Parkin (Figure 4C, Supplementary Figures 5I,J). Notably, we observed that while the TH-full promoter drives Parkin expression, there appeared to be a disruption in TH immunoreactivity within the SN. To assess any potential impact of dopaminergic neuron viability, we evaluated dopaminergic neuron marker TH by capillary-immunoblot and observed no statistically significant differences (Figure 4D, Supplementary Figures 5C,D). The Parkin protein expression in the striatum corroborated the observations in SN (Figure 4E, Supplementary Figures 5E,F). It is well reported in literature that loss of TH positive dopaminergic fibers projecting in the striatum often precedes loss of TH cell bodies in the SN. Biochemical assessment of TH in striatum showed a statistically meaningful lowering in Spark100-CAG-Parkin (human) and Spark100-TH (full)-Parkin (human) groups compared to Spark100-hSyn1-Parkin (human) group (Figure 4F, Supplementary Figures 5G,H). These data strengthened the trend observed in TH levels from SN and affirmed hSyn1 and Ef1a were the most optimal promoters in the Spark100 capsid for transducing relevant cell populations, including dopaminergic neurons.

**Figure 4:**
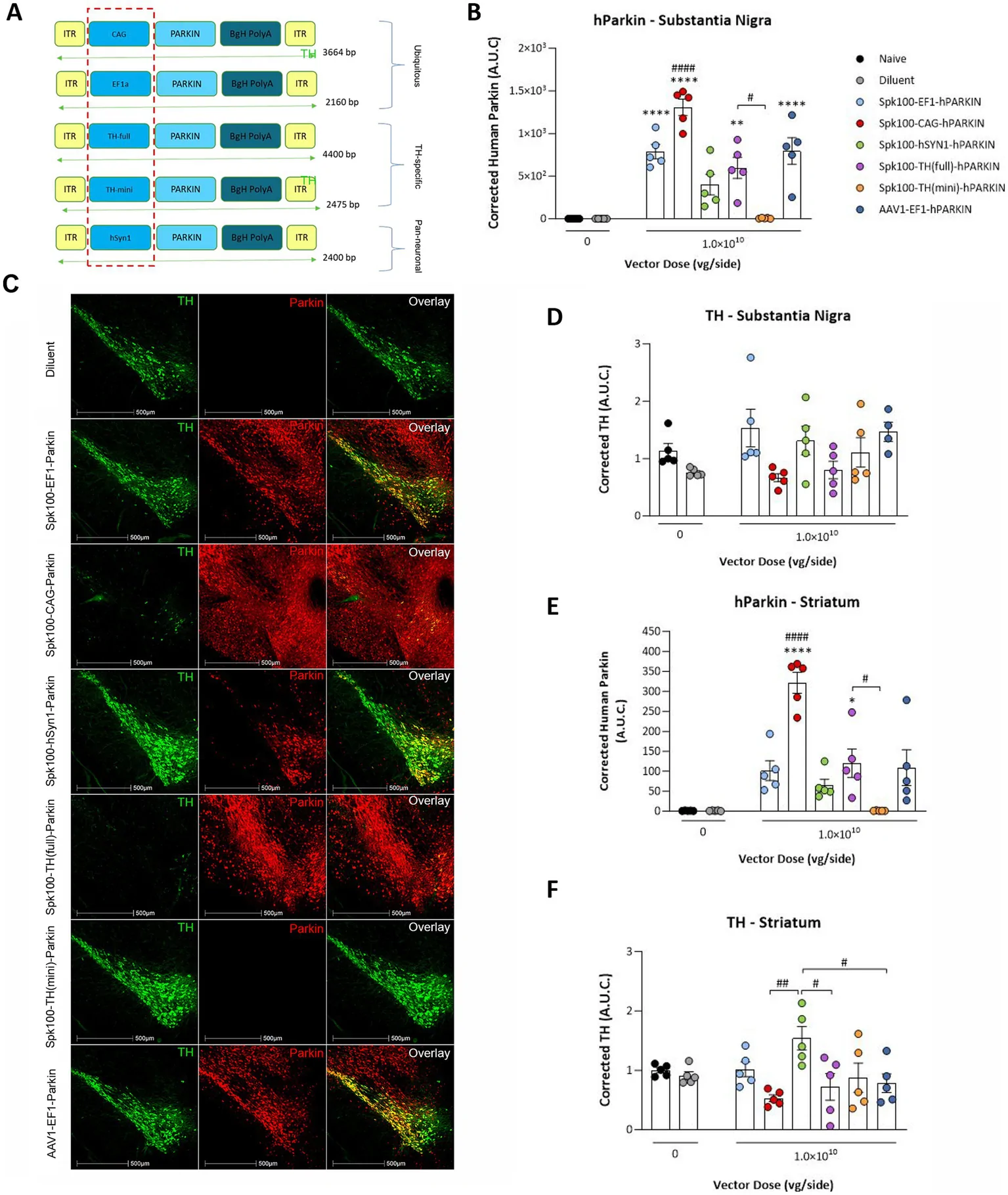
Selection of optimal promoter sequence for expressing Parkin (human) expression in dopaminergic neurons of mouse SN. **(A)** Schematic of vector constructs expressing Parkin (human) under different promoters. Dotted red box indicates variable promoter regions used for the constructs. **(B)** The normalized expression of Parkin quantified by capillary electrophoresis (human) from different promoters in SN were plotted as individual values (symbols) and group means ± SEM (bars) and analyzed by a one-way *ANOVA* followed by Tukey’s *post hoc* test. Statistically significant pairwise comparisons between the controls (naïve or diluent) and the experimental groups were reported as follows: *p < 0.05, and ***p < 0.0001. Statistically significant pairwise comparisons between experimental groups were reported as follows: ^#^*p* < 0.05, ^##^*p* < 0.01, and ^####^*p* < 0.0001 **(C)** Representative images of TH (green) and Parkin (human, red) overlay staining from animals injected with the diluent or dosed with 1.0 × 10^10^ vg/SN of Spark100-Ef1a-Parkin, Spark100-CAG-Parkin, Spark100-hSyn1-Parkin, Spark100-TH (full)-Parkin, Spark100-TH (mini)-Parkin, or AAV1-Ef1a-Parkin. **(D)** Normalized TH expression quantified by capillary electrophoresis in SN tissues following overexpression of Parkin (human) from different promoters at 1.0 × 10^10^ vg/SN. **(E,F)** Normalized expression of Parkin (human) and TH quantified by capillary electrophoresis from striatum tissues following overexpression of Parkin (human) from different promoters at 1.0 × 10^10^ vg/SN. Expression values plotted as individual values (symbols) and group means ± SEM (bars) and analyzed by a one-way ANOVA followed by Tukey’s *post hoc* test. Statistically significant pairwise comparisons between the controls (naïve or diluent) and the experimental groups were reported as follows: *p* < 0.01, and *p* < 0.0001. Statistically significant pairwise comparisons between experimental groups were reported as follows: ^#^*p* < 0.05, and ^####^*p* < 0.0001. SNpc, substantia nigra pars compacta; TH, tyrosine hydroxylase; vg, vector genome.

Based on the prior observations from Figures 3E,F that high Parkin expression may result in adverse glial activation, we sought to determine whether there were promoter and expression-mediated alterations in astrocyte (Gfap) and microglial (Iba1) immunoreactivity in this study. Indeed, we observed the ubiquitous CAG promoter driving the highest levels of Parkin protein expression in the SN also resulted in significant upregulation in Iba1 (Figure 5A, *p* < 0.0001, Supplementary Figures 6A,B) and Gfap (Figure 5B, *p* < 0.0001, Supplementary Figures 6C,D). Consistently, we observed reduced TH expression in SN and Striatum following Parkin expression from the ubiquitous CAG promoter. Importantly, all other constructs tested did not display any significant alterations in glial immunoreactivity (representative images Figure 5C). These results confirm expression of Parkin is feasible in dopaminergic neurons and well-tolerated at shorter time-points, but suggest the importance of careful dose-finding studies.

**Figure 5:**
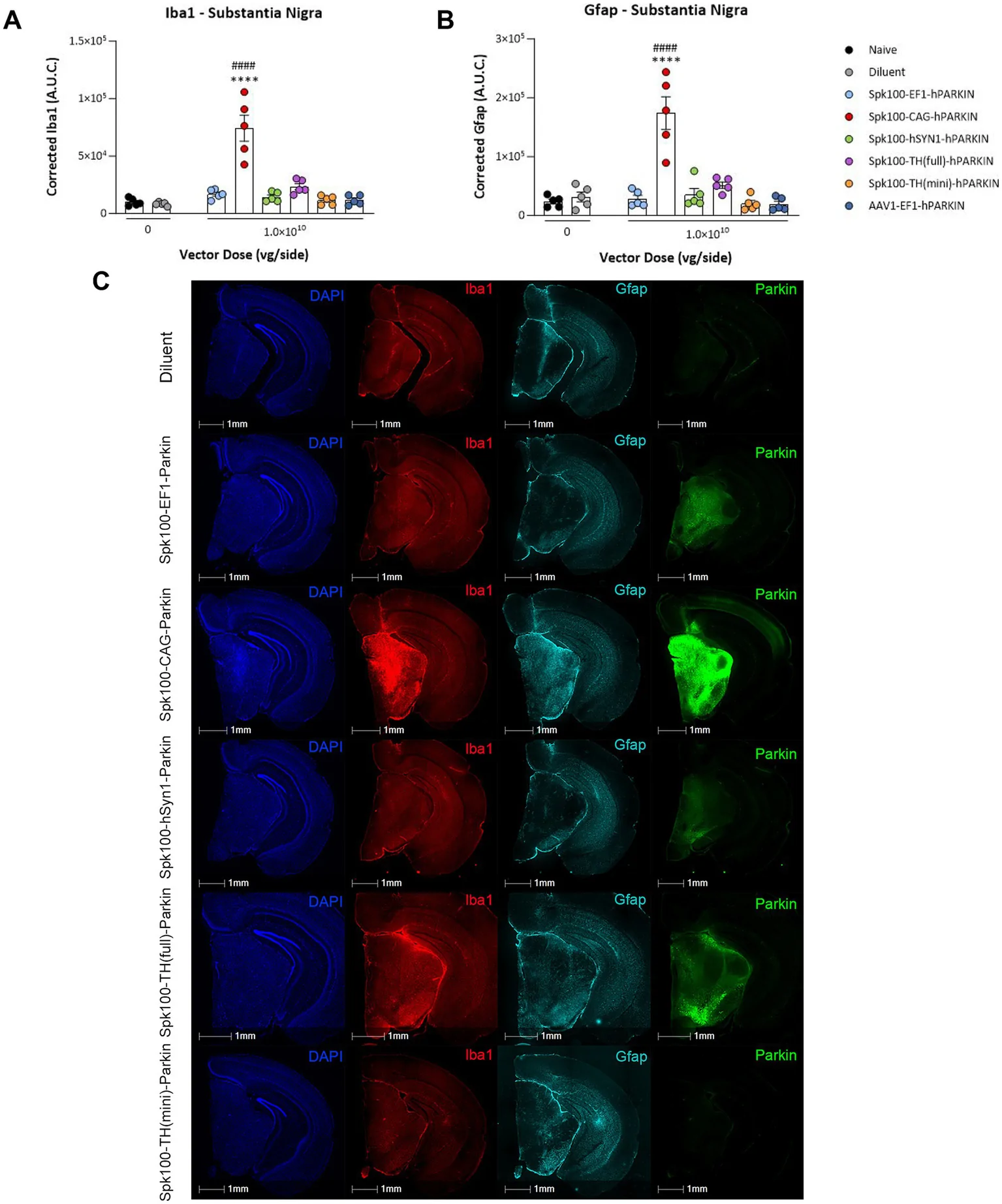
Evaluation of gliosis markers Iba1 and Gfap in mouse SN tissues following overexpression of Parkin (human) from different promoters. **(A,B)** Normalized Iba1 and Gfap expression quantified by capillary electrophoresis in SN tissues following overexpression of Parkin (human) from different promoters were plotted as individual values (symbols) and group means ± SEM (bars) and analyzed by a one-way ANOVA followed by Tukey’s *post hoc* test. **(C)** Representative images of DAPI (blue), Iba1 (red), Gfap (cyan), and human Parkin (green) staining from animals injected with the diluent or dosed with 1.0 × 10^10^ vg/SN of Spark100-Ef1a-Parkin, Spark100-CAG-Parkin, Spark100-hSyn1-Parkin, Spark100-TH (full)-Parkin, Spark100-TH (mini)-Parkin. Statistically significant pairwise comparisons between the controls (naïve or diluent) and the experimental groups were reported as follows: ****p < 0.0001. Statistically significant pairwise comparisons between experimental groups were reported as follows: ^####^*p* < 0.0001.

## 4 Discussion

Our research provides a foundation for the development of AAV-Parkin gene therapy as a treatment for early-onset Parkinson’s disease (EOPD) caused by biallelic loss of function mutation in *PRKN*. A key advantage of gene therapy is its ability to target specific cells, which in this case allows for the direct delivery of functional Parkin to relevant cell types including dopaminergic cells of the substantia nigra. This precise delivery is crucial for restoring proper clearance of damaged mitochondria via mitophagy, a process hypothesized to be critical for slowing the progression of dopaminergic neuron loss.

We have demonstrated that replenishing functional Parkin successfully restores pUb^Ser65^ defects in a range of *in vitro* models, including patient-derived cells. Furthermore, our *in vivo* studies establish a feasible and well-tolerated strategy for the targeted expression of Parkin specifically within SN- an approach gaining significant traction due to its demonstrated effectiveness in treating conditions like aromatic L-amino acid decarboxylase (AADC) deficiency (Pearson et al., 2021).

### 4.1 Developed a model to study Parkin activation and mitophagy initiation *in vitro*

A critical aspect of our study was our reinforcing pUb^Ser65^ as a reliable marker of PINK1-Parkin pathway activation (Koyano et al., 2014; Kane et al., 2024; Kazlauskaite et al., 2014). By inducing mitochondrial stress with FCCP, we demonstrated that Parkin KO neuroblastoma cells exhibit a deficiency in pUb^Ser65^ amplification, a deficit functionally rescued by the introduction of AAV-Parkin. The observed MOI-dependent increase in pUb^Ser65^, coupled with the reduction in Parkin protein levels upon stress, supports the notion that AAV-delivered Parkin actively engages with and is degraded alongside dysfunctional mitochondria. While this degradation provides an indirect assessment of downstream cargo clearance, we acknowledge that it does not directly demonstrate completed mitophagic flux. Future studies utilizing dynamic flux reporters will be highly valuable to fully map the kinetics of mitochondrial turnover following AAV-Parkin administration. Crucially, the direct visualization of pUb^Ser65^ co-localizing with mitochondria following FCCP treatment in Parkin KO cells treated with AAV-Parkin provides confirmation of its functional recruitment (Figure 1).

Extending these findings to EOPD patient-derived fibroblasts with diverse *PRKN* mutations reinforces the translational relevance of our work. The observation of diminished pUb^Ser65^ amplification in patient cells under valinomycin-induced mitochondrial stress, and its subsequent restoration with AAV-Parkin, demonstrates that our gene therapy approach can restore Parkin function in the setting of diverse variants (Figure 2). While our current findings center on restoring the PINK1-Parkin axis, it is important to contextualize this within the broader network of mitochondrial quality control. As noted, the loss of mitochondrial membrane potential initiates the clearance of uncoupled mitochondria through both Parkin-dependent and Parkin-independent mitophagy pathways. Parkin-independent mechanisms, which often rely on receptor-mediated proteins (such as FUNDC1, BNIP3, and NIX/BNIP3L) (Terešak et al., 2022) or alternative E3 ligases, can directly recruit autophagosomes to damaged mitochondria, bypassing the necessity for Parkin-mediated ubiquitin chain amplification. The interplay between these parallel pathways likely accounts for the basal mitochondrial clearance and the residual pUb^Ser65^ generation observed in our Parkin-deficient models.

### 4.2 Demonstrated transgene expression and short-term tolerability in mammalian models

Demonstrating successful transgene expression and tolerability in a mammalian model is paramount for gene therapy development. For our initial *in vivo* feasibility and tolerability studies in rats, we selected the AAV1 capsid. AAV1 was chosen due to its well-characterized and highly efficient tropism for brain tissues, as well as its historical precedent in driving robust transgene expression following direct intraparenchymal central nervous system delivery (Wang et al., 2019; Blits et al., 2010). Our rat study confirmed the feasibility of AAV-Parkin (human) delivery to the SN via intraparenchymal injection, achieving dose-dependent human Parkin expression that co-localized effectively with TH-positive dopaminergic neurons (Figure 3B). Quantitatively, the 3e8 vg/SN dose approximated our targeted range of 50–100% of endogenous levels (averaging ~135%). While this serves as a crucial proof-of-concept for therapeutic replacement strategies, the notable variability between animals indicates that precise physiological restoration remains challenging. At the 3e9 vg/SN and 3e10 vg/SN doses, we achieved Parkin overexpression significantly beyond physiological levels (Supplementary Figure 4H). We observed some variability in Parkin expression (Figures 3C,D). This could be because we were targeting a small deep brain structure like the SN, but it did not affect the study’s overall conclusion. Additionally, this variability is largely inherent to the mechanical limitations of rodent stereotaxic surgery, where delivering micro-volumes into a minute, deep brain structure like the SNpc is highly susceptible to sub-millimeter deviations in needle placement and vector spread. However, this preclinical challenge is substantially mitigated in the clinical setting; modern human gene therapy trials utilize real-time intraoperative MRI guidance and Convection-Enhanced Delivery (CED). These advanced techniques allow for active monitoring of the infusion cloud, ensuring highly precise target coverage and robust clinical reproducibility (Pearson et al., 2021).

While doses below 3e10 vg/SN were well-tolerated, the highest dose resulted in significant upregulation of glial activation markers (Gfap and Iba1), as well as a reduction in striatal dopamine and impaired dopamine turnover, indicative of dopaminergic neuronal dysfunction (Figures 3E–H). Although a vector-only control at this highest dose was not included, the correlation between supraphysiological Parkin expression and adverse glial activation strongly suggests excessive Parkin levels, rather than just viral load, can induce inflammatory response. This underscores the delicate balance required for effective gene therapy and highlights the necessity of precise dose-finding to avoid unintended consequences, even with a protective factor like Parkin. From a translational perspective, our study was designed to assess the toxicity of the intact therapeutic article (vector and transgene together), as empty capsids are not typically utilized as controls in late-stage IND-enabling studies. Furthermore, our subsequent promoter study robustly demonstrated that capsid load alone does not drive this toxicity. However, we acknowledge that the current data demonstrate short-term tolerability, and these findings are not sufficient to draw definitive conclusions regarding long-term CNS safety. Future studies evaluating long-term safety and efficacy in larger animal models will be essential before this approach can be advanced to the clinic.

### 4.3 Promoter selection for optimal targeting

In parallel with the rat study, we conducted a complementary study in mice to select the optimal promoter for our proprietary Spark100 capsid, aiming for the best transduction of target cell populations. As demonstrated in our promoter evaluation, the proprietary Spark100 capsid showed similar transduction efficiency of dopaminergic neurons in the SN compared to the commonly used AAV1 capsid when both utilized the Ef1a promoter (Figure 4C). Furthermore, proprietary internal evaluations confirmed that Spark100 performs comparably to AAV1 and AAV9 in this target tissue (Supplementary Figure 2). A dose of 1e10 vg/SN was selected for this study to account for the differential strengths of the promoters used (hSyn1 is known to drive weaker expression than the CAG promoter). The objective was to ensure sufficient human Parkin expression from all promoters at the given dose and time point, thereby facilitating an informed interpretation regarding the optimal promoter selection.

The CAG promoter drove Parkin to supraphysiological levels of overexpression, causing significant gliosis (Figures 5A–C). While we did not see a significant reduction in TH levels in the SN and striatum compared to control animals, there was a consistent trend. This difference was only statistically meaningful when we compared Spark100-CAG-Parkin (human) to Spark100-hSyn1-Parkin (human) in the striatum (Figure 4E). Unlike the rat study (Figure 3), the mouse study had additional groups injected with vectors using Spark100 capsid at 1e10vg/SN (Figure 4B). Since these groups showed no signs of gliosis (based on Iba1 and Gfap analysis) compared to diluent and naïve groups, we can conclude that the significant gliosis seen in the Spark100-CAG-Parkin group was caused by the supraphysiological levels of human Parkin, and not by titers/dose of the capsid.

Other tested promoters were determined to be less optimal for more nuanced reasons. For instance, the TH promoter initially appeared to be an ideal candidate for expression in TH-positive neurons. However, we observed a trend of reduced TH protein expression in dopaminergic neurons by immunohistochemistry (Figure 4C), without any associated upregulation of inflammatory markers. Ultimately, the hSyn1 and Ef1a promoters demonstrated the most ideal expression level and specificity for Parkin gene therapy, without any glial inflammation.

While these findings collectively lay a foundation for a viable therapeutic approach for Parkin-PD—and underscore the need for dose-finding studies to optimize Parkin expression levels for clinical translation—we acknowledge certain limitations. We prioritized assessing efficacy in patient-derived human cells over *in vivo* rodent models. This is primarily because currently available *PRKN* knockout rodent models do not exhibit the overt nigrostriatal degeneration or motor deficits seen in EOPD patients. *PRKN* knockout conditions do not fully recapitulate the diverse structural and functional consequences of human *PRKN* mutations. Sporadic PD models, such as alpha-synuclein transgenics, MPTP (1-methyl-4-phenyl-1,2,3,6-tetrahydropyridine)-treatment, or preformed fibril models, although valuable, are driven by pathological mechanisms that do not accurately capture the specific monogenic nature of Parkin deficiency (Hioki et al., 2026). In addition, our findings build upon and refine previous proof-of-concept studies in the field of *PRKN*-associated Parkinson’s disease gene therapy. While recent efforts have successfully demonstrated the neuroprotective potential of Parkin overexpression—often utilizing standard AAV serotypes to drive ubiquitous, high-level expression (Hioki et al., 2026)—many of these approaches have primarily focused on establishing efficacy. In contrast, our current study prioritizes the translational hurdles of vector optimization and overexpression related short-term tolerability. By systematically evaluating our proprietary Spark100 capsid and rigorously testing how different regulatory elements (e.g., Ef1a, hSyn1, CAG, and TH promoters) influence both target transduction and local tissue tolerability, our approach addresses the safety bottlenecks required for clinical advancement. This aligns with a growing consensus in the broader gene therapy field that successful CNS therapeutics require precise regulation to restore physiological function without triggering the dose-dependent inflammatory responses we observed with the CAG promoter. Human PRKN mutations are highly diverse, ranging from whole-exon deletions to missense mutations that generate unstable or catalytically inactive proteins (Menon et al., 2024). Because the presence of an endogenous mutant protein could theoretically alter cellular machinery or interfere with therapeutic rescue, demonstrating efficacy in a single engineered knockout line is insufficient. The observation of diminished pUb^Ser65^ amplification across 8–9 patient lines under valinomycin-induced stress, and its subsequent restoration with AAV-Parkin, demonstrates that our gene replacement strategy successfully overrides the endogenous mutant background.

Since the preclinical dataset presented in this manuscript strictly focused on establishing a path for monogenic Parkin-PD, we prioritized a patient-specific *in vitro* approach with distinct mutations to demonstrate mechanistic rescue via early pathway activation. We acknowledge that the current study did not evaluate downstream mitochondrial functional rescue, such as ATP production, respiration, or ROS clearance. Evaluating these metabolic endpoints will be a critical next step in future efficacy studies to fully characterize the cellular benefits of AAV-Parkin therapy. We utilized rodent models strictly to evaluate vector tropism, tolerability, and candidate optimization. Ultimately, these limitations do not diminish the central finding: an approximation of physiological restoration of Parkin is achievable and demonstrates short-term tolerability, provided that careful dose titration and promoter selection are employed.

## Data Availability

All data has been provided in the main text or supplementary section of this article, with no data deposited to external repositories. All associated data presented here are available on reasonable request from the corresponding authors (SB and M-GB).

## Author disclaimer

The contributions of the NIH author(s), AG and DN were made as part of their official duties as NIH federal employees, are in compliance with agency policy requirements, and are considered Works of the United States Government. However, the findings and conclusions presented in this paper are those of the author(s) and do not necessarily reflect the views of the NIH or the United States Department of Health and Human Services.
