# Supplementary material for: Restoring Parkin function: an AAV gene therapy approach for early-onset Parkinson’s disease
Source: Front Mol Neurosci. 2026 Oct 21;19:1939065. doi: 10.3389/fnmol.2026.1939065 (PMC13731301; doi:10.3389/fnmol.2026.1939065)
Supplement: Supplementary file 1 [file Data_Sheet_1.pdf]

Restoring Parkin Function: An AAV Gene Therapy Approach for Early-Onset Parkinson's Disease

Supplemental data file

Supplementary Figure 1: Identification of Phospho-ubiquitin serine 65 (pUb^{Ser65}) as a marker of Parkin-mediated mitophagy:

A

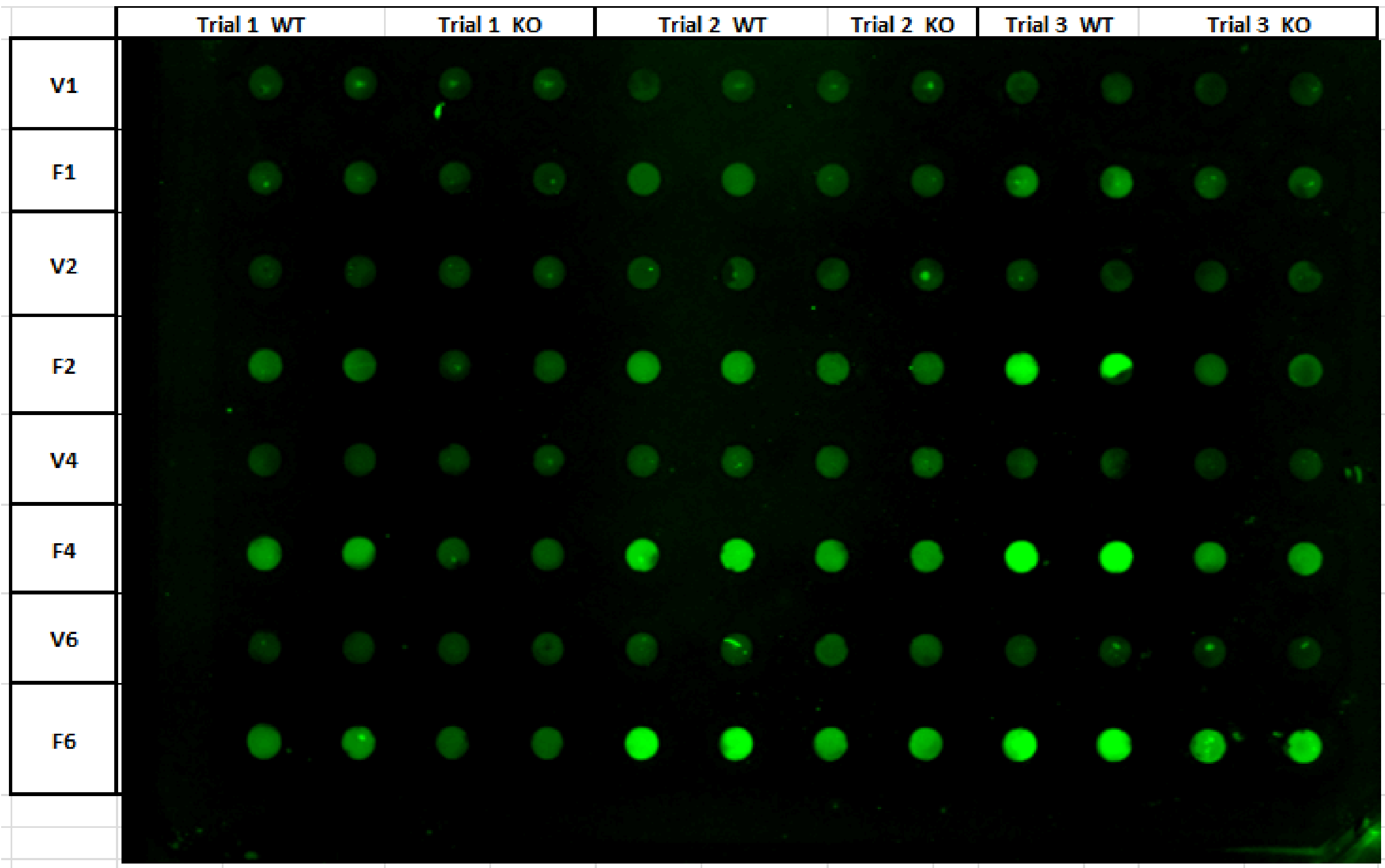

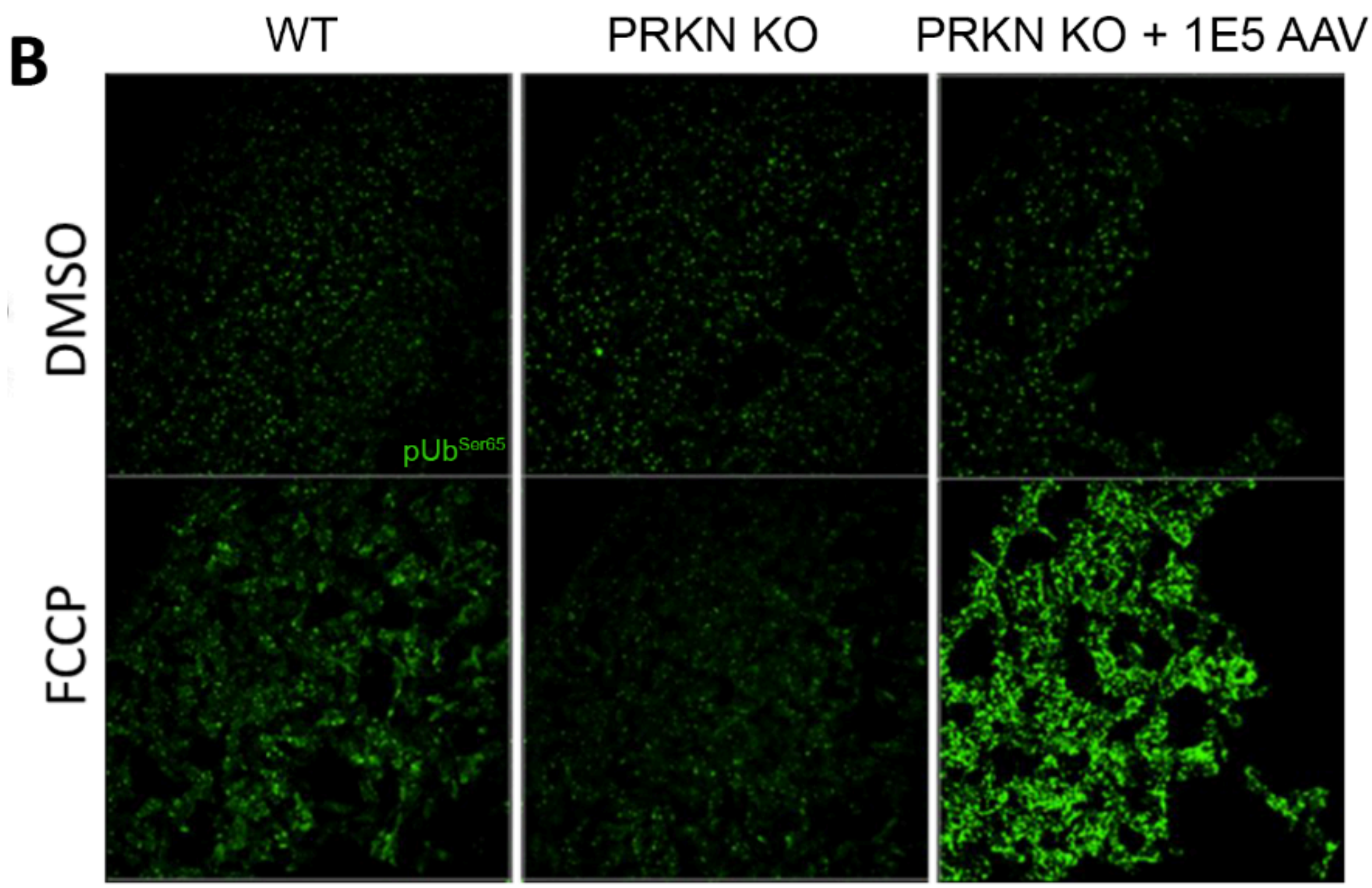

A) Dot blot image measuring pUb^{Ser65} response in WT and Parkin KO SH-SY5Y cell lines. Across 3 trials, FCCP treatment triggered a significant upregulation in pUb^{Ser65} response across a 6-hour time course in WT. B) Representative immunocytochemistry staining visually confirming pUb^{Ser65} signal at MOI 1e5.

Supplementary Figure 2: Successful transduction of TH-positive dopaminergic neurons within the murine SN was achieved using AAV1, AAV9, and Spark100-based viral vectors:

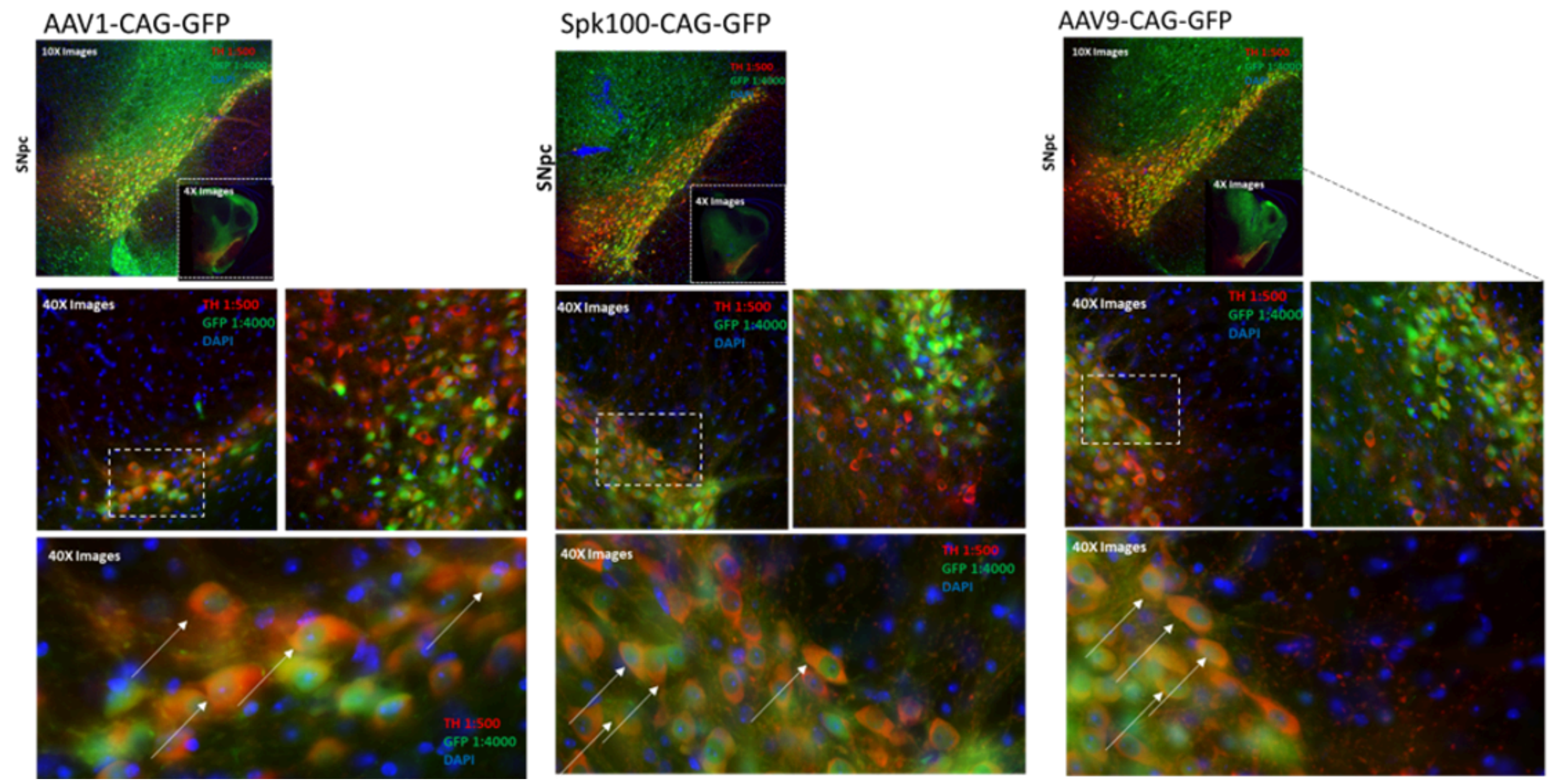

AAV1,AAV9 and AAV-Spark100 vectors successfully transduced TH-positive dopaminergic neurons of the SN of wild-type mice. Representative images of TH, GFP, DAPI staining of animal SN-injected with 1.00×10^{10} vg/side of AAV1-CAG-GFP,**AAV-CAG-Spark100 and** AAV1-CAG-GFP vector. Photographs of the regions of interest were taken with 4x (A, E), 10x (B, F), and 40x (C, G) objectives.The arrowheads in panels indicate successfully transduced TH-positive dopaminergic neurons.

GFP = green fluorescent protein; SN = substantia nigra; TH = tyrosine hydroxylase, vg = vector genome.

Supplementary Figure 3: Selection of AAV2 capsid using CAG-GFP and validation of AAV2-CAG-Parkin (human) restoring pUb^{Ser65} signal in PD patient fibroblasts:

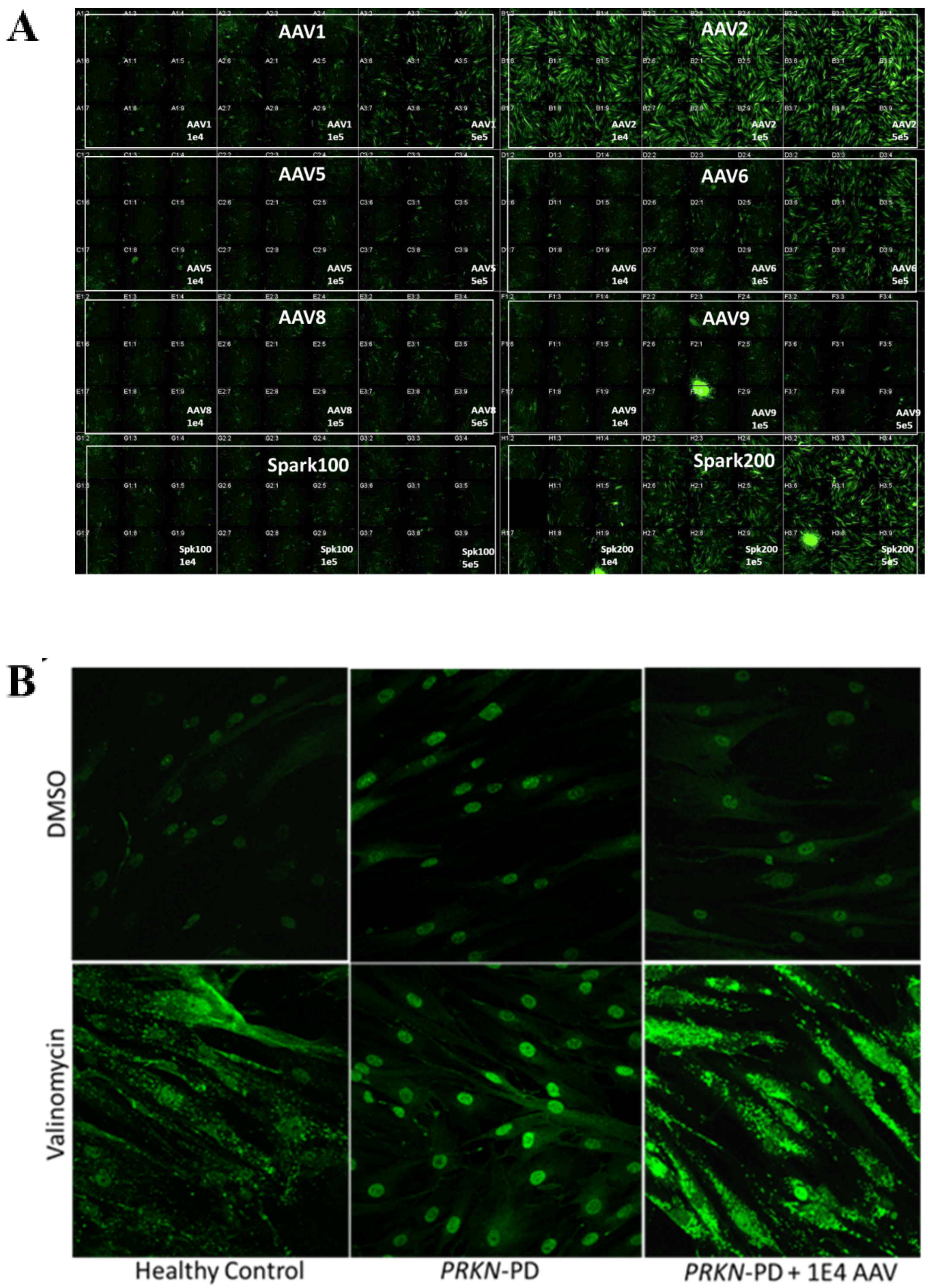

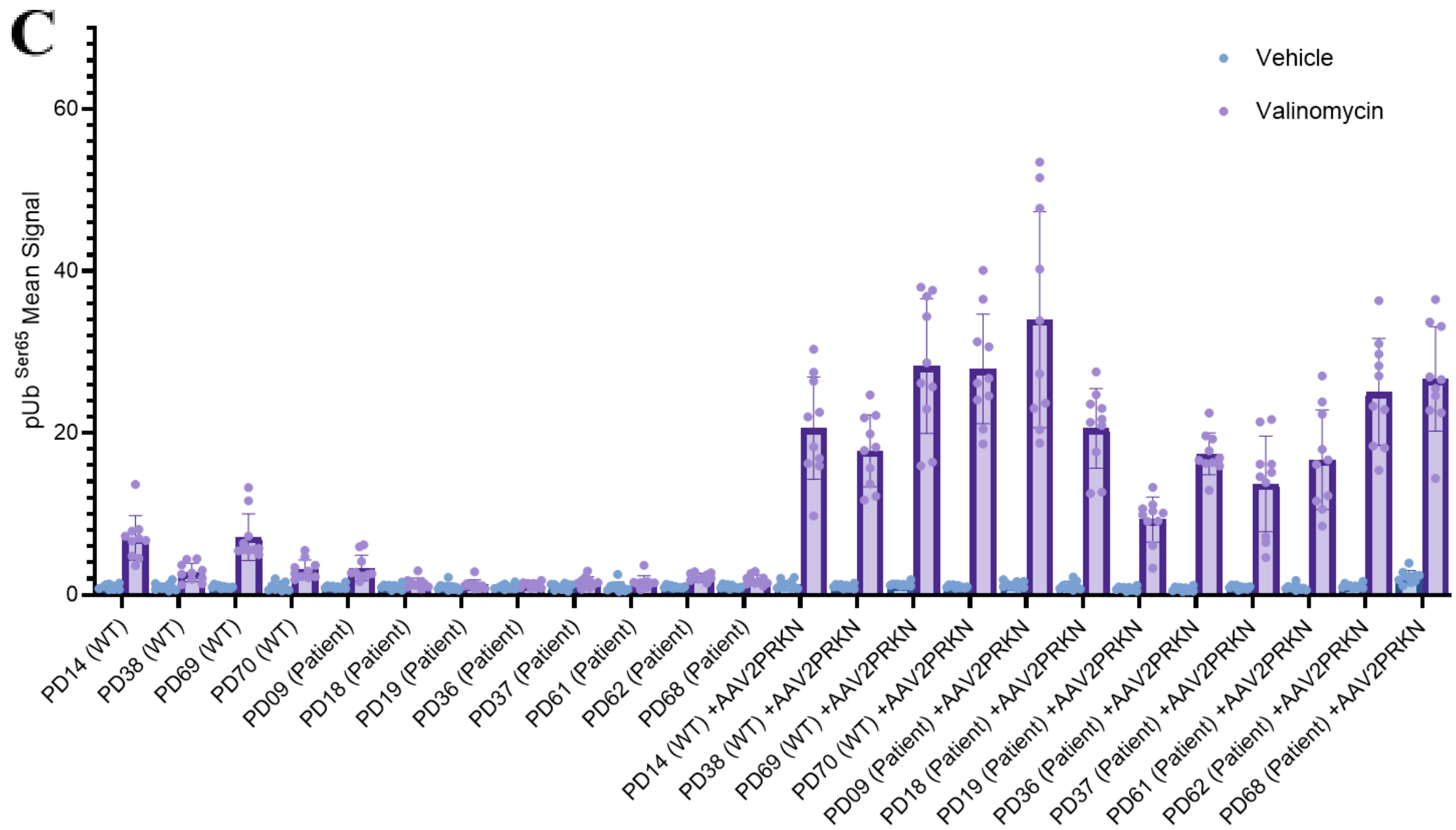

A) Representative images of WT human skin fibroblasts tested with various AAV capsids expressing GFP. B) Representative images of pUb^{Ser65} staining in PD patient-derived fibroblasts showing restoration of mitophagy when cells are treated with AAV2-CAG-Parkin and stressed with valinomycin C) Quantification of pUb^{Ser65} fluorescence mean signal intensity from each Healthy control and PD patient lines, with and without valinomycin treatment.

Supplementary Figure 4. Dose-dependent Parkin (human) expression following stereotaxic delivery of AAV-Ef1a-Parkin (human) in Rat substantia nigra pars compacta (SNpc):

A

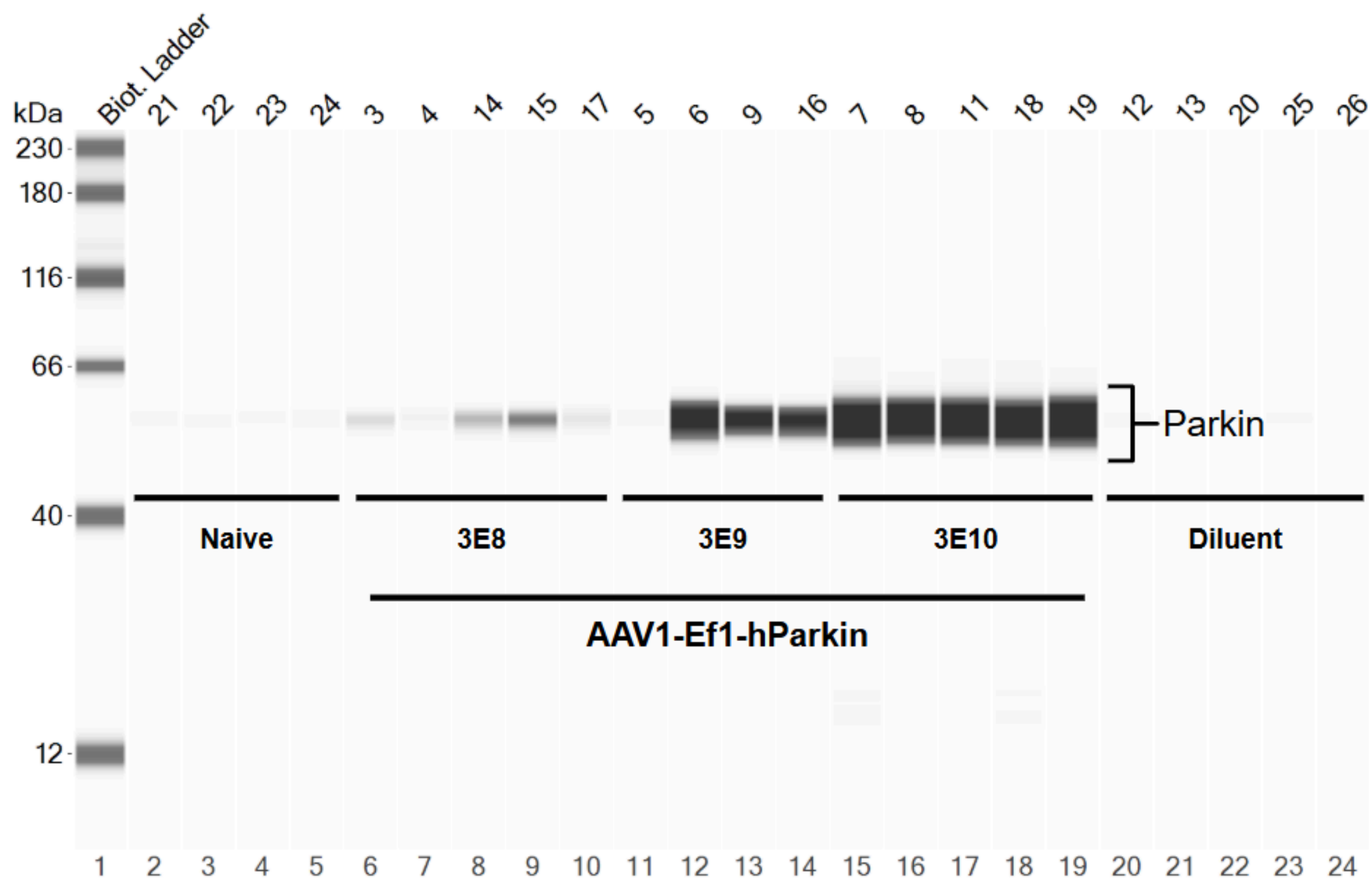

B

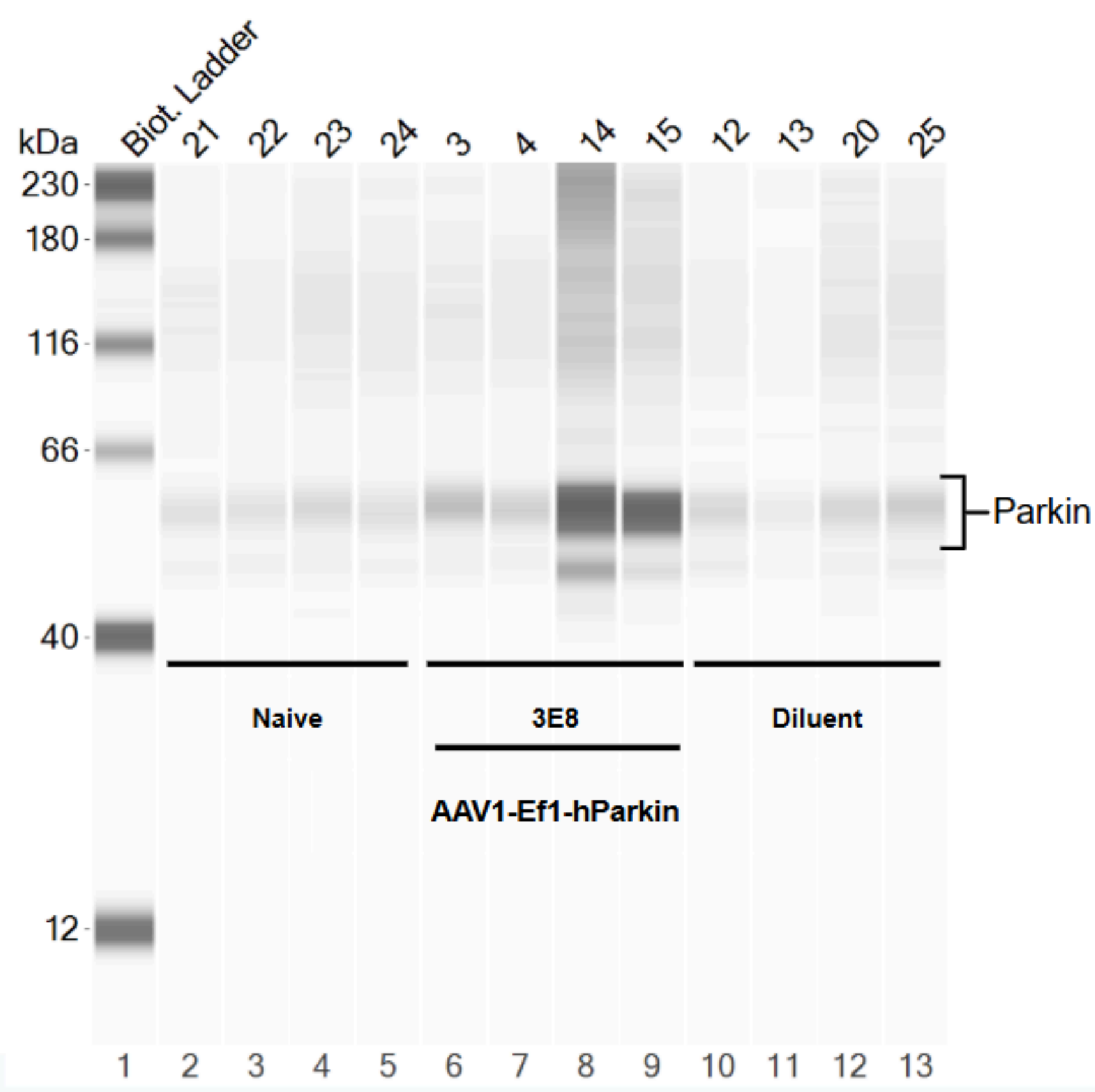

C

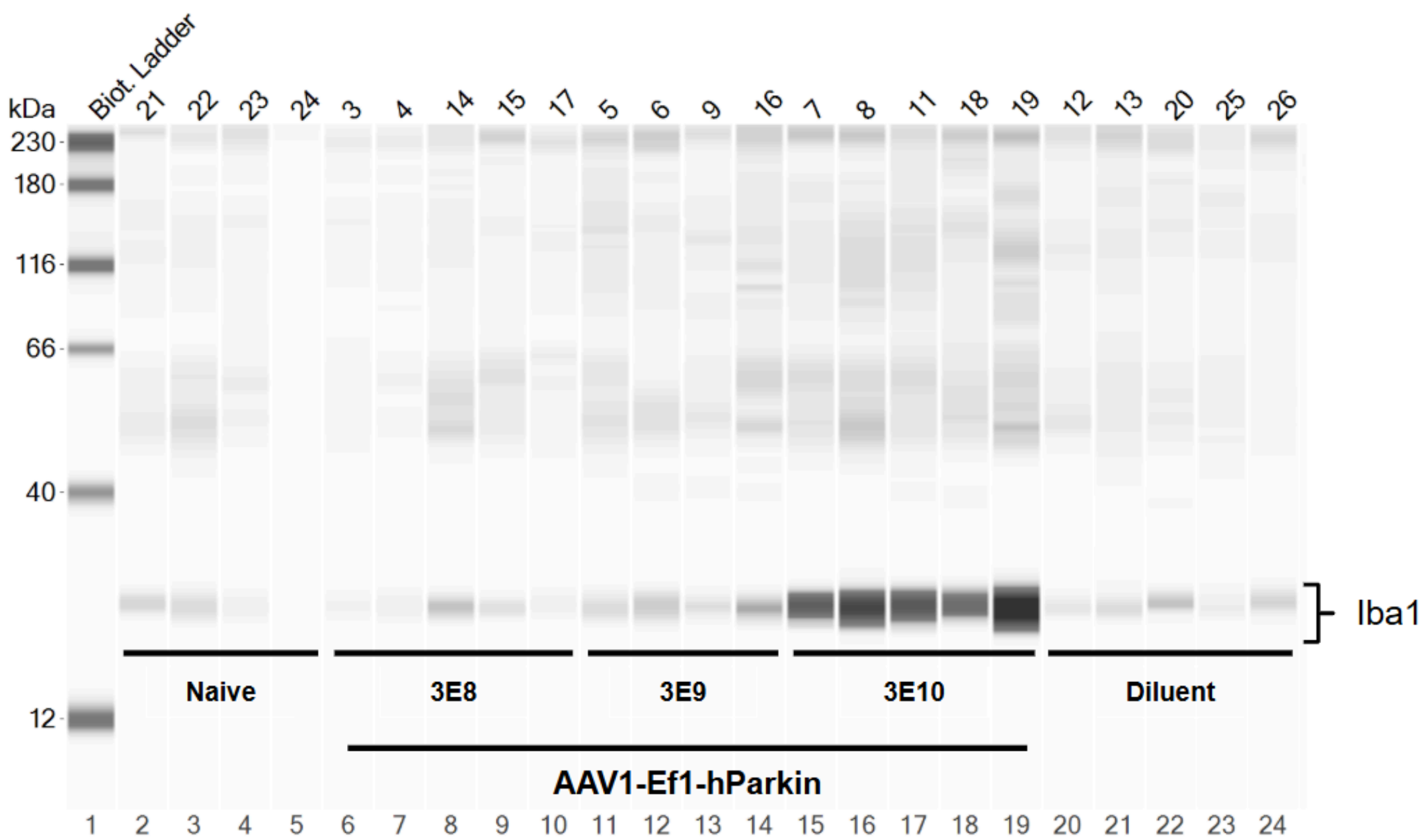

kDa
Biot. Ladder
21 22 23 24 3 4 14 15 17 5 6 9 16 7 8 11 18 19 12 13 20 25 26
230
180
116
66
40
12
Iba1
Naive
3E8
3E9
3E10
Diluent
AAV1-Ef1-hParkin
1 2 3 4 5 6 7 8 9 10 11 12 13 14 15 16 17 18 19 20 21 22 23 24

D

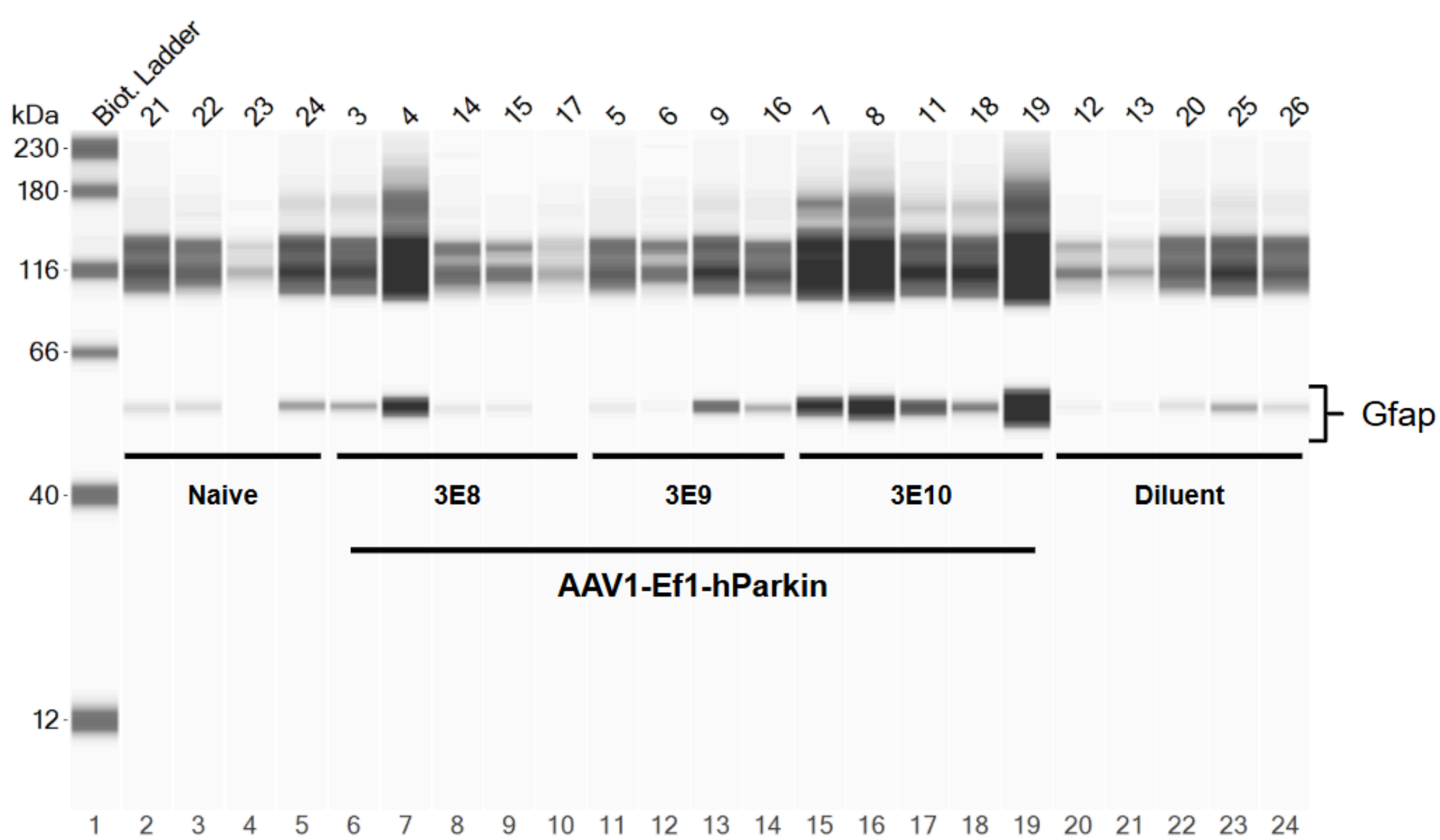

kDa
Biot. Ladder
21 22 23 24 3 4 14 15 17 5 6 9 16 7 8 11 18 19 12 13 20 25 26
230
180
116
66
40
12
Gfap
Naive
3E8
3E9
3E10
Diluent
AAV1-Ef1-hParkin
1 2 3 4 5 6 7 8 9 10 11 12 13 14 15 16 17 18 19 20 21 22 23 24

E

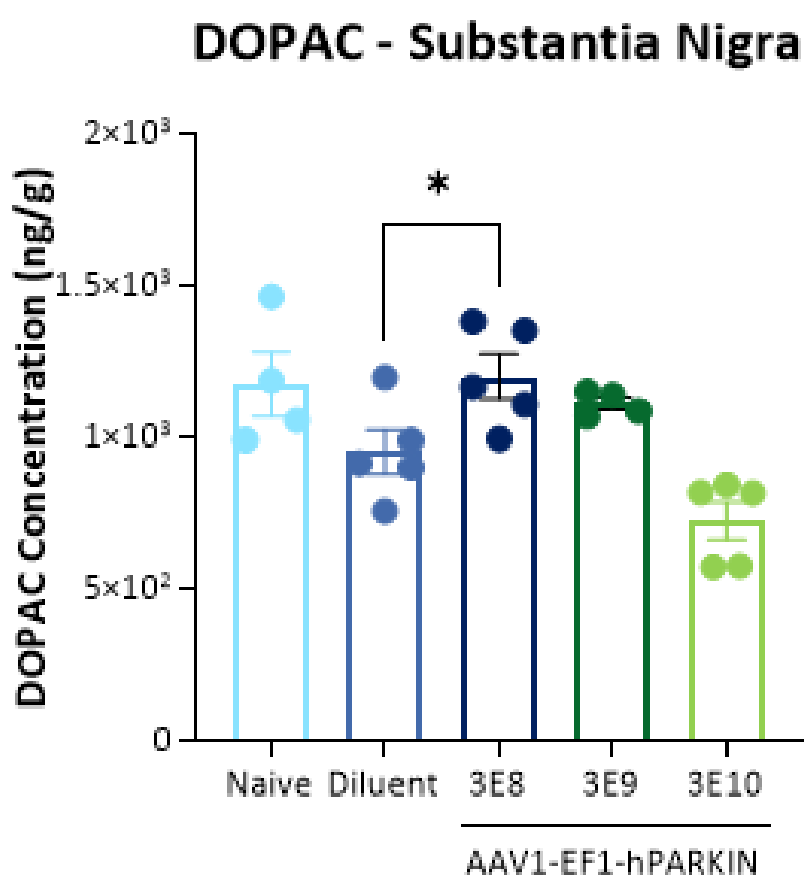

DOPAC - Substantia Nigra
DOPAC Concentration (ng/g)
2×10³
1.5×10³
1×10³
5×10²
0
*
Naive
Diluent
3E8
3E9
3E10
AAV1-EF1-hPARKIN

F

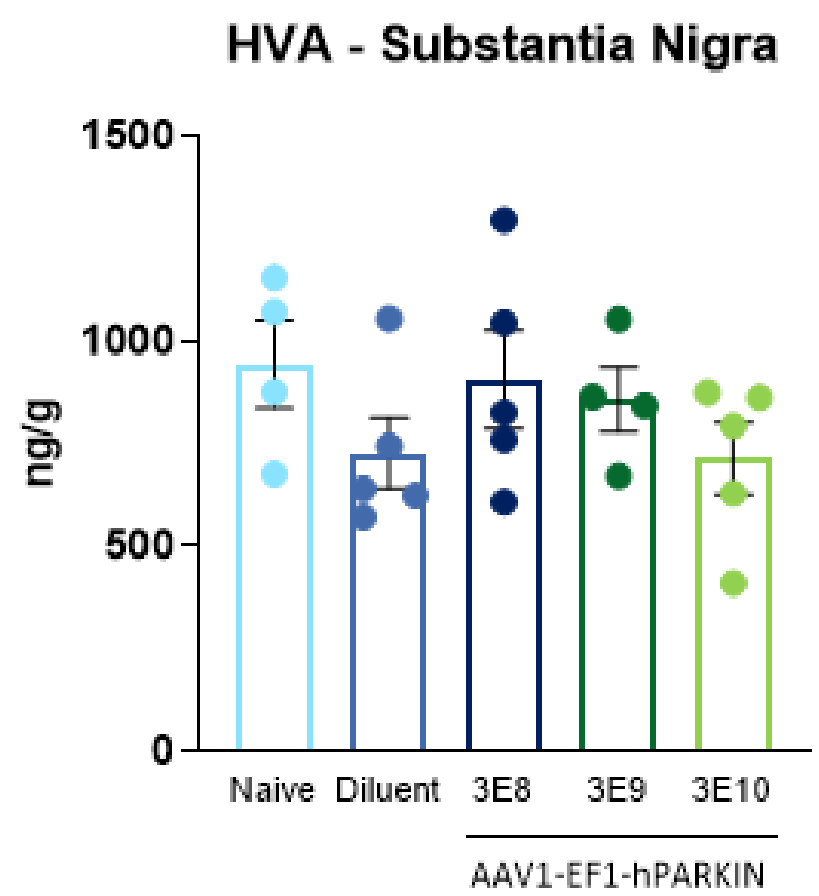

HVA - Substantia Nigra
ng/g
1500
1000
500
0
Naive
Diluent
3E8
3E9
3E10
AAV1-EF1-hPARKIN

G

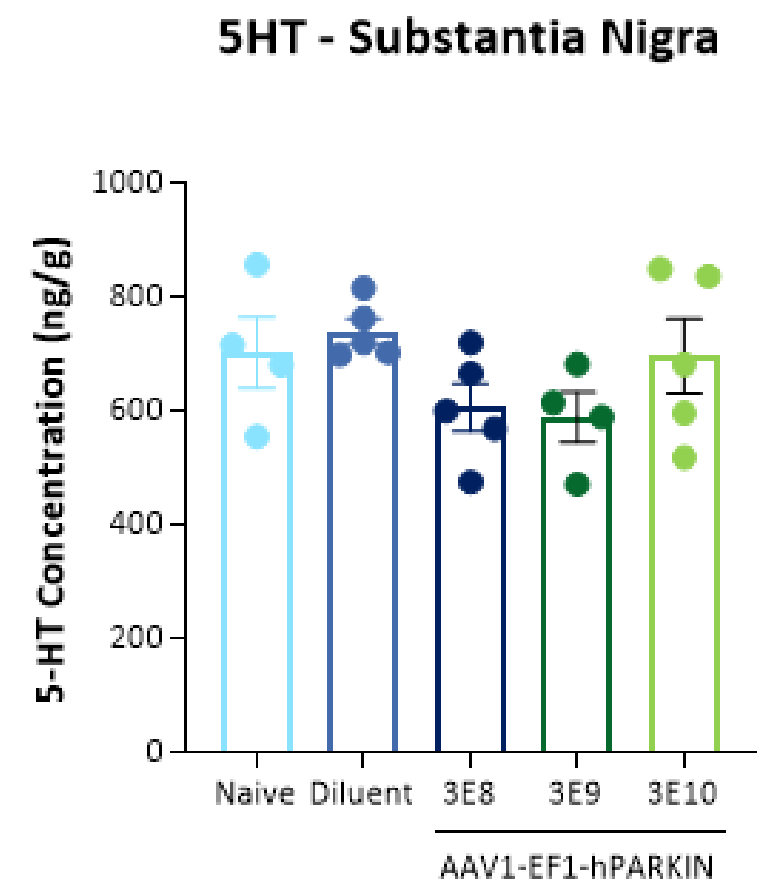

5HT - Substantia Nigra
5-HT Concentration (ng/g)
1000
800
600
400
200
0
Naive
Diluent
3E8
3E9
3E10
AAV1-EF1-hPARKIN

H

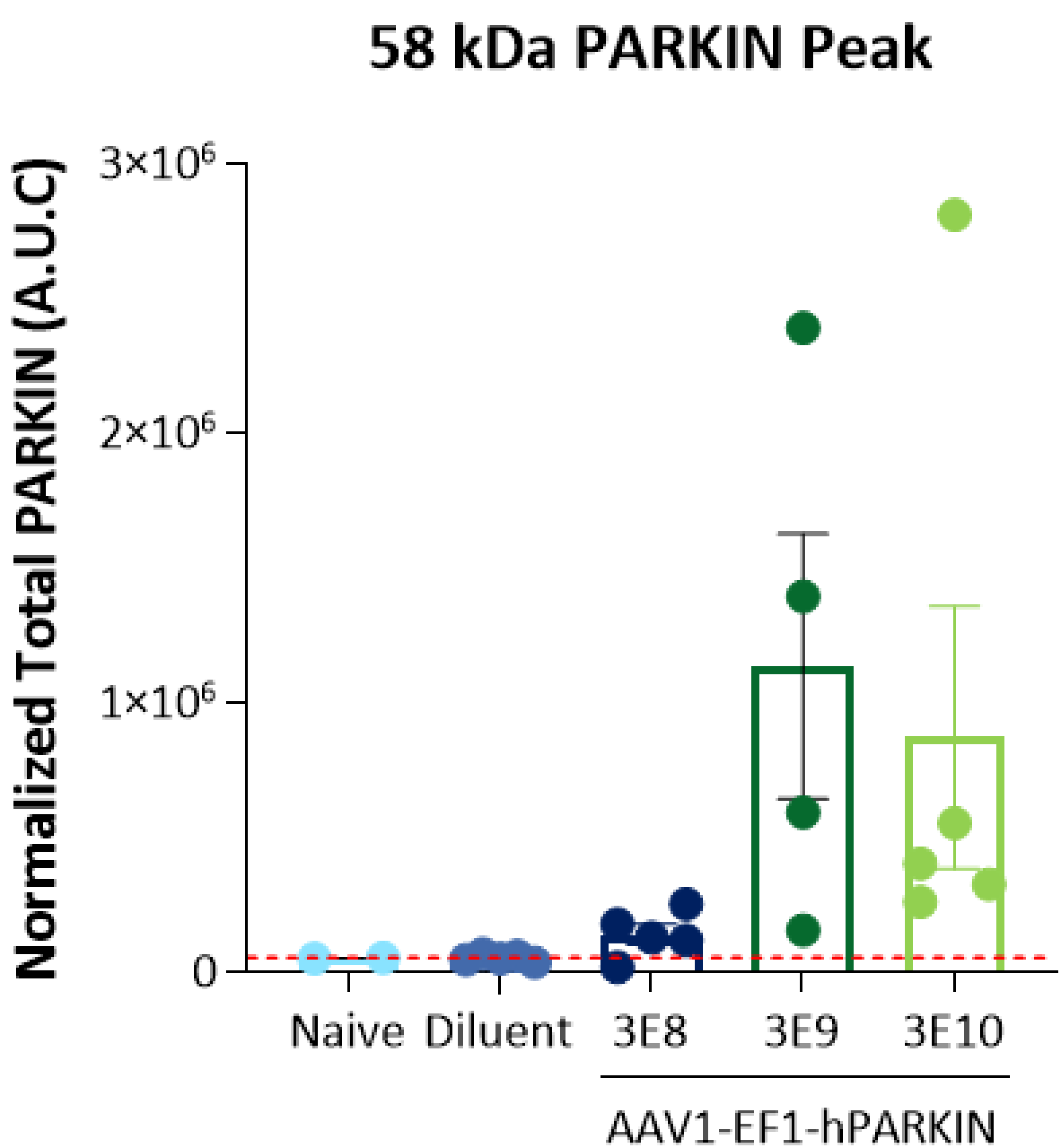

58 kDa PARKIN Peak
Normalized Total PARKIN (A.U.C)
3×10⁶
2×10⁶
1×10⁶
0
Naive
Diluent
3E8
3E9
3E10
AAV1-EF1-hPARKIN

Capillary electrophoresis images showing expression of human Parkin (A), Total Parkin (B) Iba1 (C) and Gfap(D) from SN tissues of individual animals following stereotaxic delivery of AAV-Ef1a-Parkin (human) in Rat substantia nigra pars compacta (SNpc) (E-G) HPLC for dopamine metabolites 3,4-dihydroxyphenylacetic acid (DOPAC), homovanillic acid (HVA) and 5-HT, *p=0.0344 Ordinary one-way ANOVA with Dunnett's multiple comparisons test. (H) Quantified 58KDa Parkin band detected with PRK8 antibody that detects both human and endogenous rat Parkin under the tested conditions

Supplementary Figure 5: Optimal Promoter selection for expressing Parkin(human) Expression in dopaminergic neurons of mouse SN.

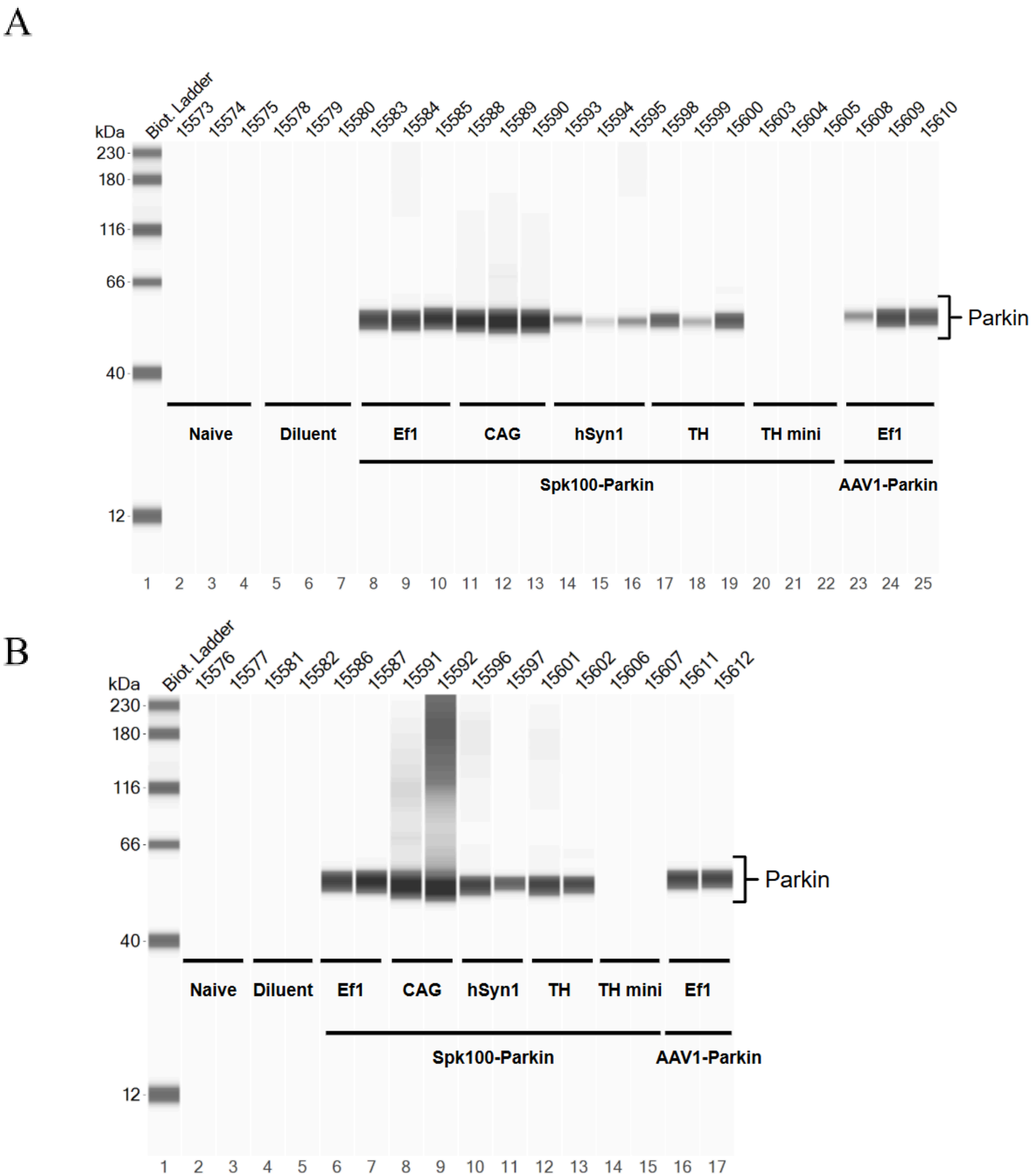

C

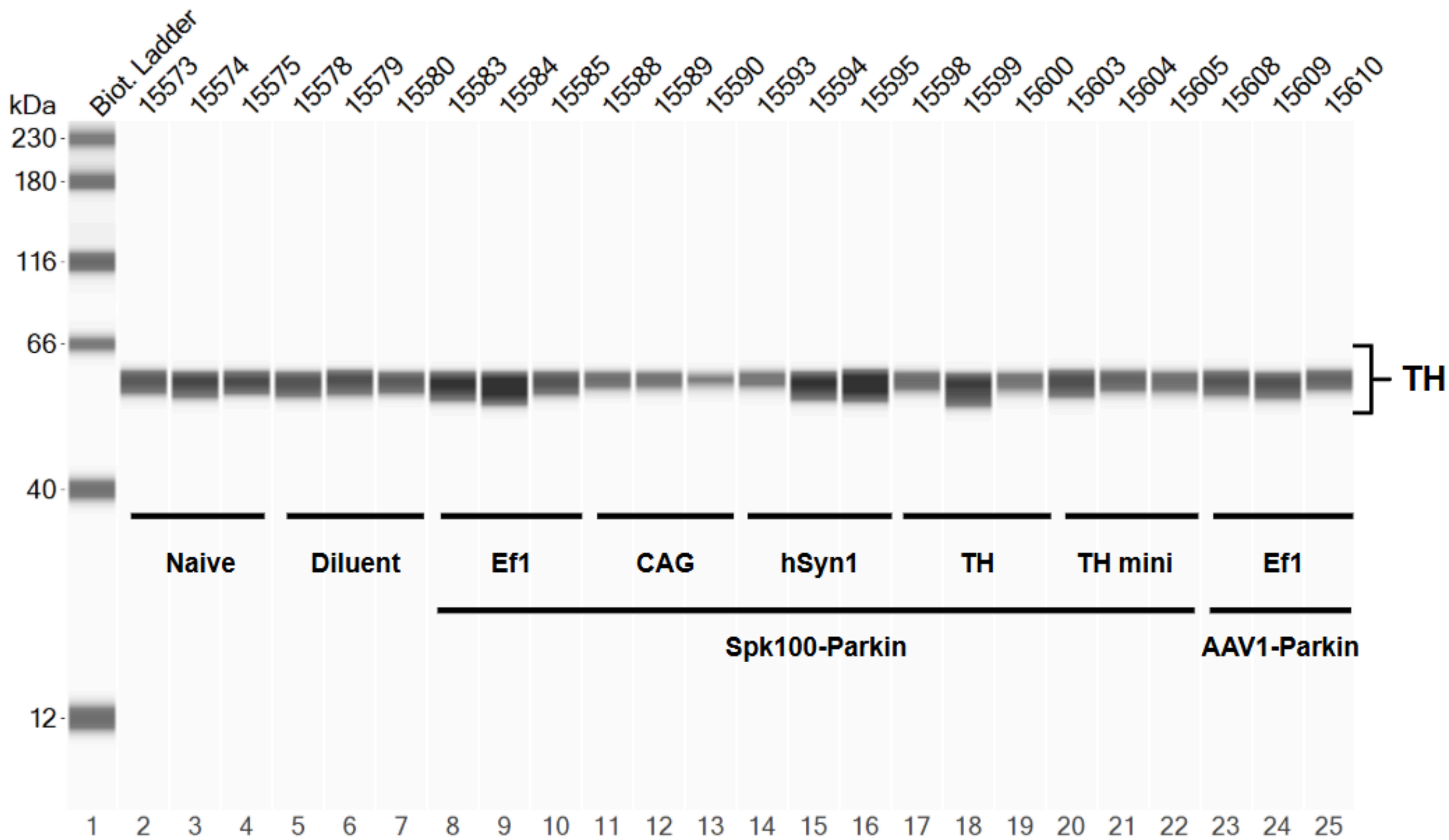

Biot. Ladder
15573
15574
15575
15578
15579
15580
15583
15584
15585
15588
15589
15590
15593
15594
15595
15598
15599
15600
15603
15604
15605
15608
15609
15610
kDa
230
180
116
66
40
12
TH
Naive
Diluent
Ef1
CAG
hSyn1
TH
TH mini
Ef1
Spk100-Parkin
AAV1-Parkin
1 2 3 4 5 6 7 8 9 10 11 12 13 14 15 16 17 18 19 20 21 22 23 24 25

D

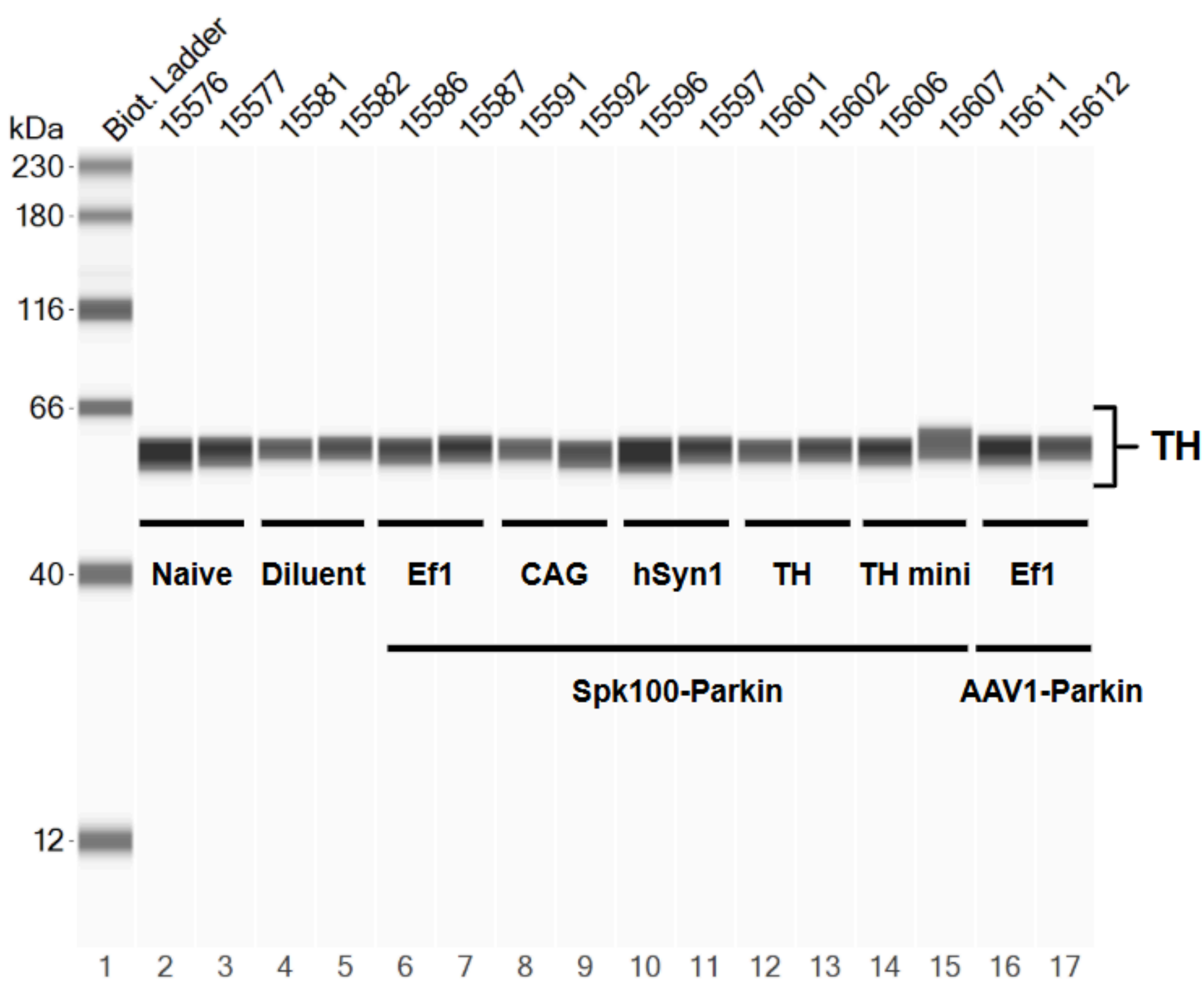

Biot. Ladder
15576
15577
15581
15582
15586
15587
15591
15592
15596
15597
15601
15602
15606
15607
15611
15612
kDa
230
180
116
66
40
12
TH
Naive
Diluent
Ef1
CAG
hSyn1
TH
TH mini
Ef1
Spk100-Parkin
AAV1-Parkin
1 2 3 4 5 6 7 8 9 10 11 12 13 14 15 16 17

E

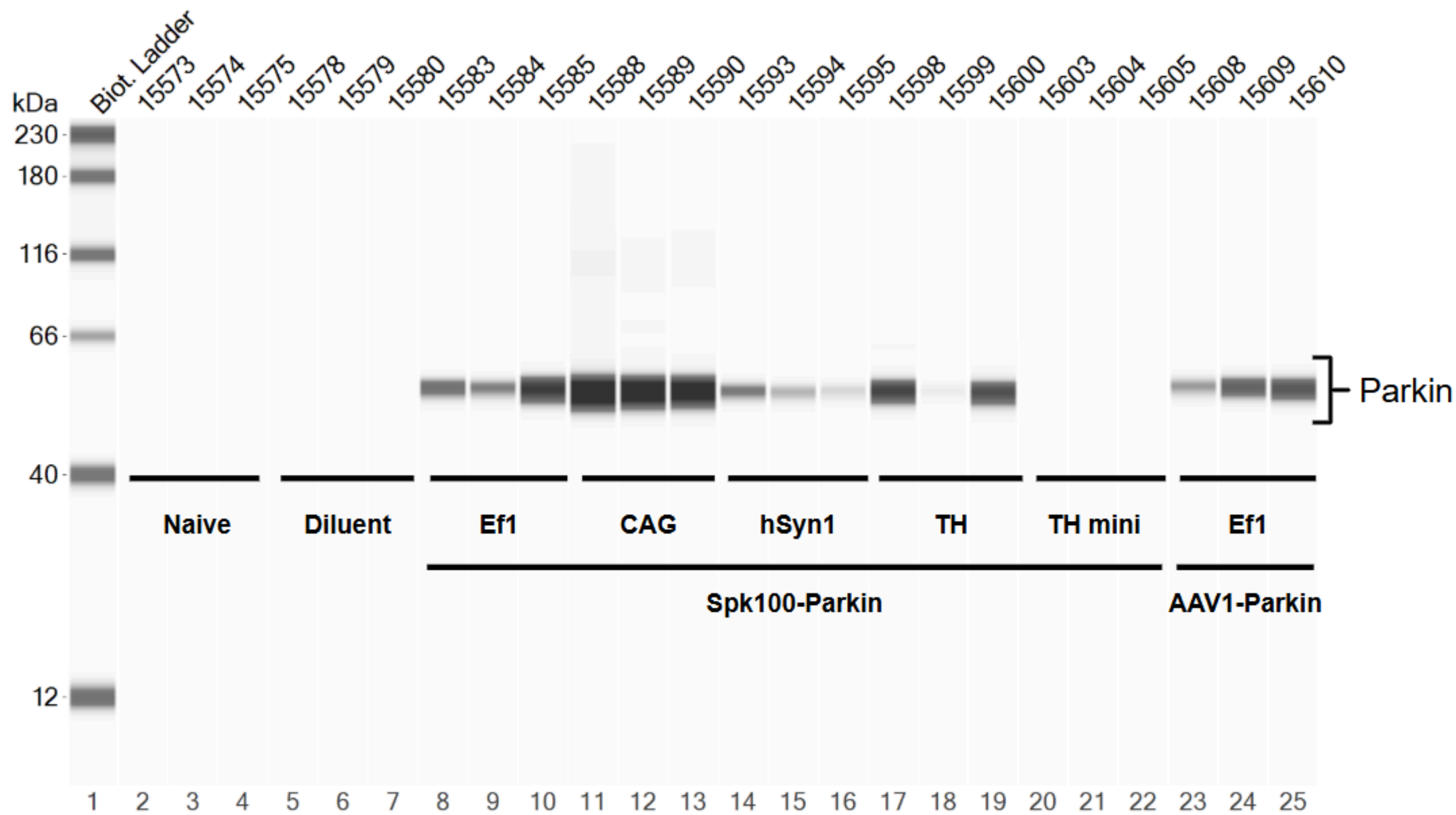

kDa
Biot. Ladder
15573
15574
15575
15578
15579
15580
15583
15584
15585
15588
15589
15590
15593
15594
15595
15598
15599
15600
15603
15604
15605
15608
15609
15610
230
180
116
66
40
12
Parkin
Naive
Diluent
Ef1
CAG
hSyn1
TH
TH mini
Ef1
Spk100-Parkin
AAV1-Parkin
1 2 3 4 5 6 7 8 9 10 11 12 13 14 15 16 17 18 19 20 21 22 23 24 25

F

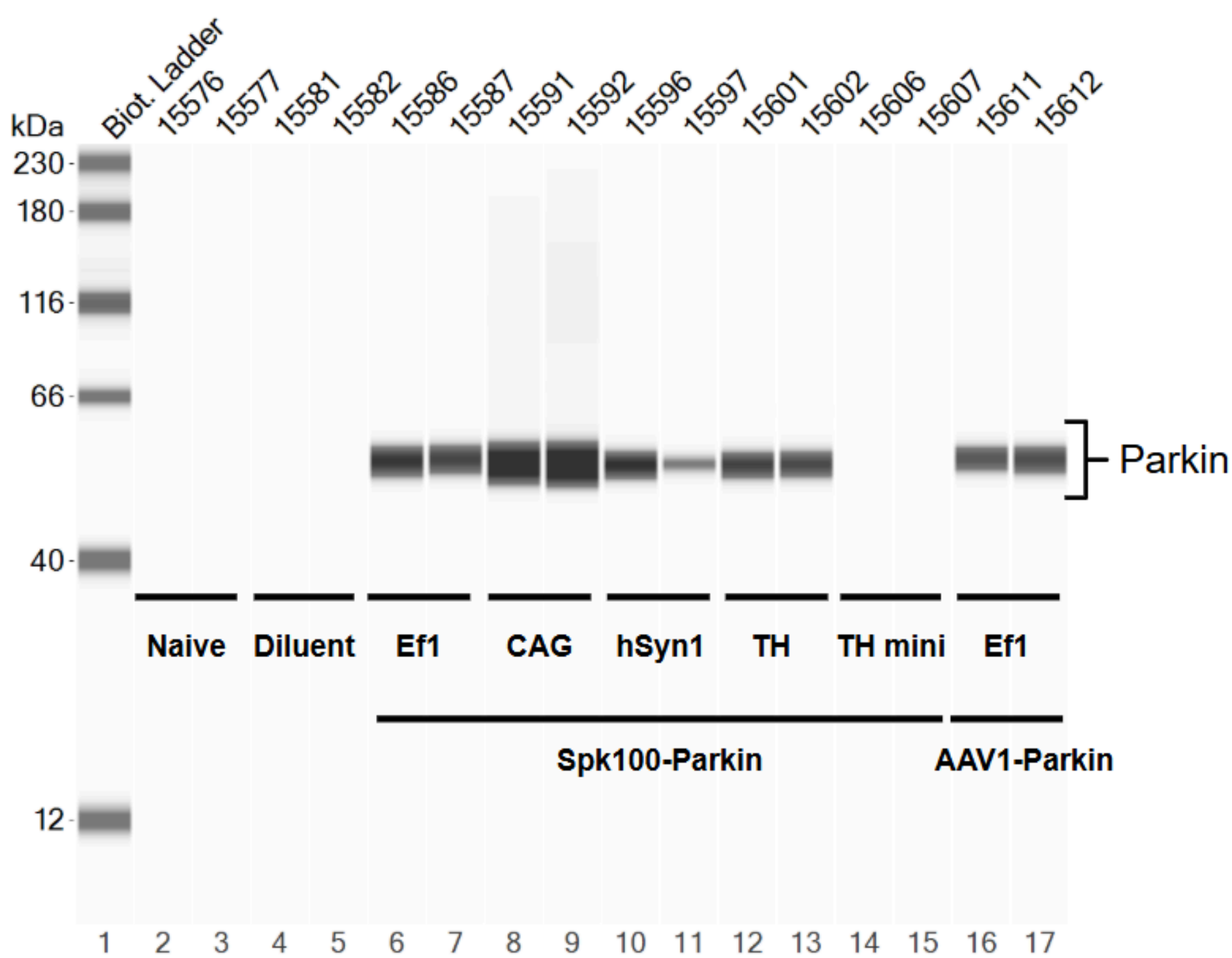

kDa
Biot. Ladder
15576
15577
15581
15582
15586
15587
15591
15592
15596
15597
15601
15602
15606
15607
15611
15612
230
180
116
66
40
12
Parkin
Naive
Diluent
Ef1
CAG
hSyn1
TH
TH mini
Ef1
Spk100-Parkin
AAV1-Parkin
1 2 3 4 5 6 7 8 9 10 11 12 13 14 15 16 17

G

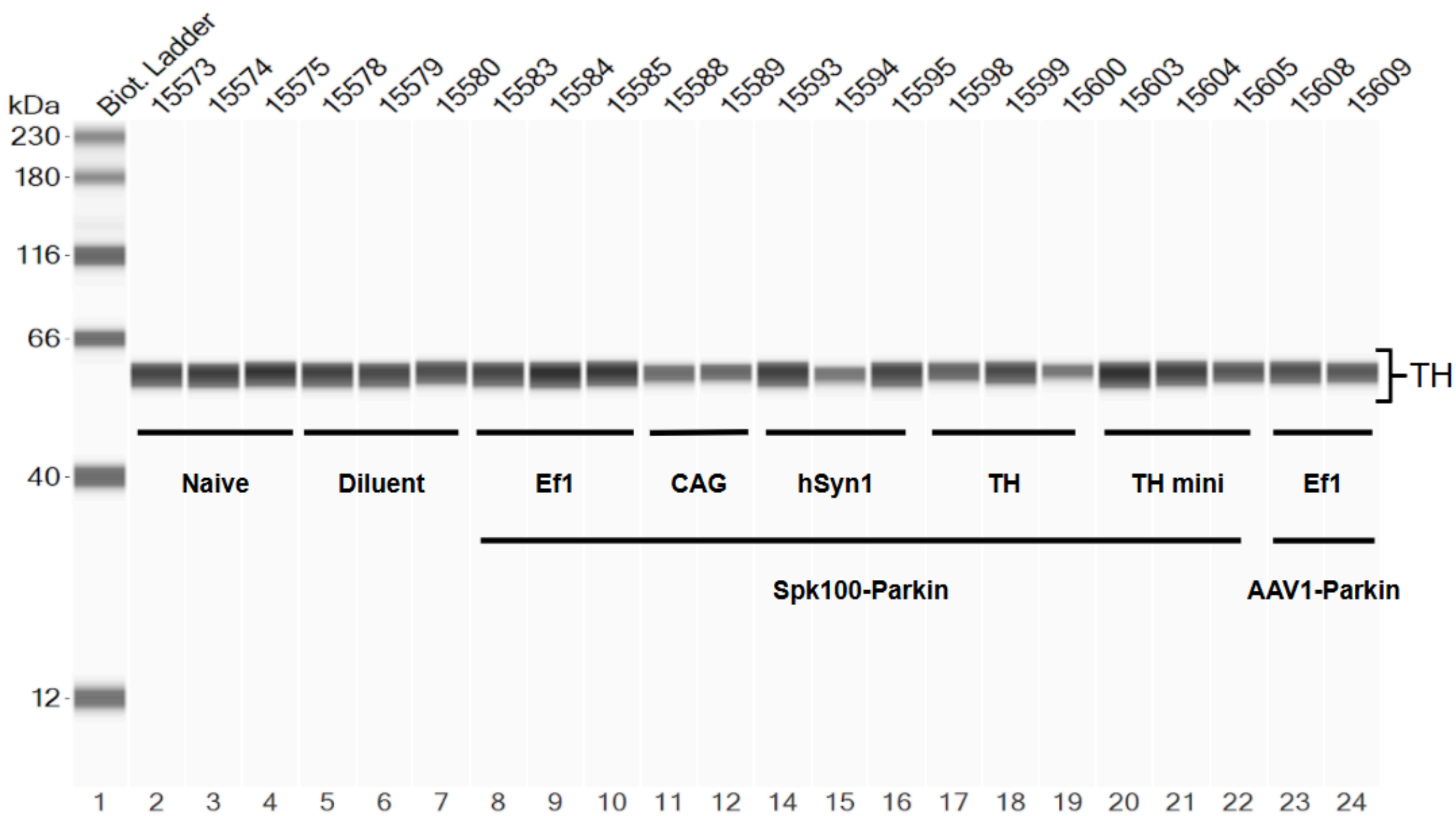

Biot. Ladder
15573
15574
15575
15578
15579
15580
15583
15584
15585
15588
15589
15593
15594
15595
15598
15599
15600
15603
15604
15605
15608
15609
kDa
230
180
116
66
40
12
TH
Naive
Diluent
Ef1
CAG
hSyn1
TH
TH mini
Ef1
Spk100-Parkin
AAV1-Parkin
1 2 3 4 5 6 7 8 9 10 11 12 14 15 16 17 18 19 20 21 22 23 24

H

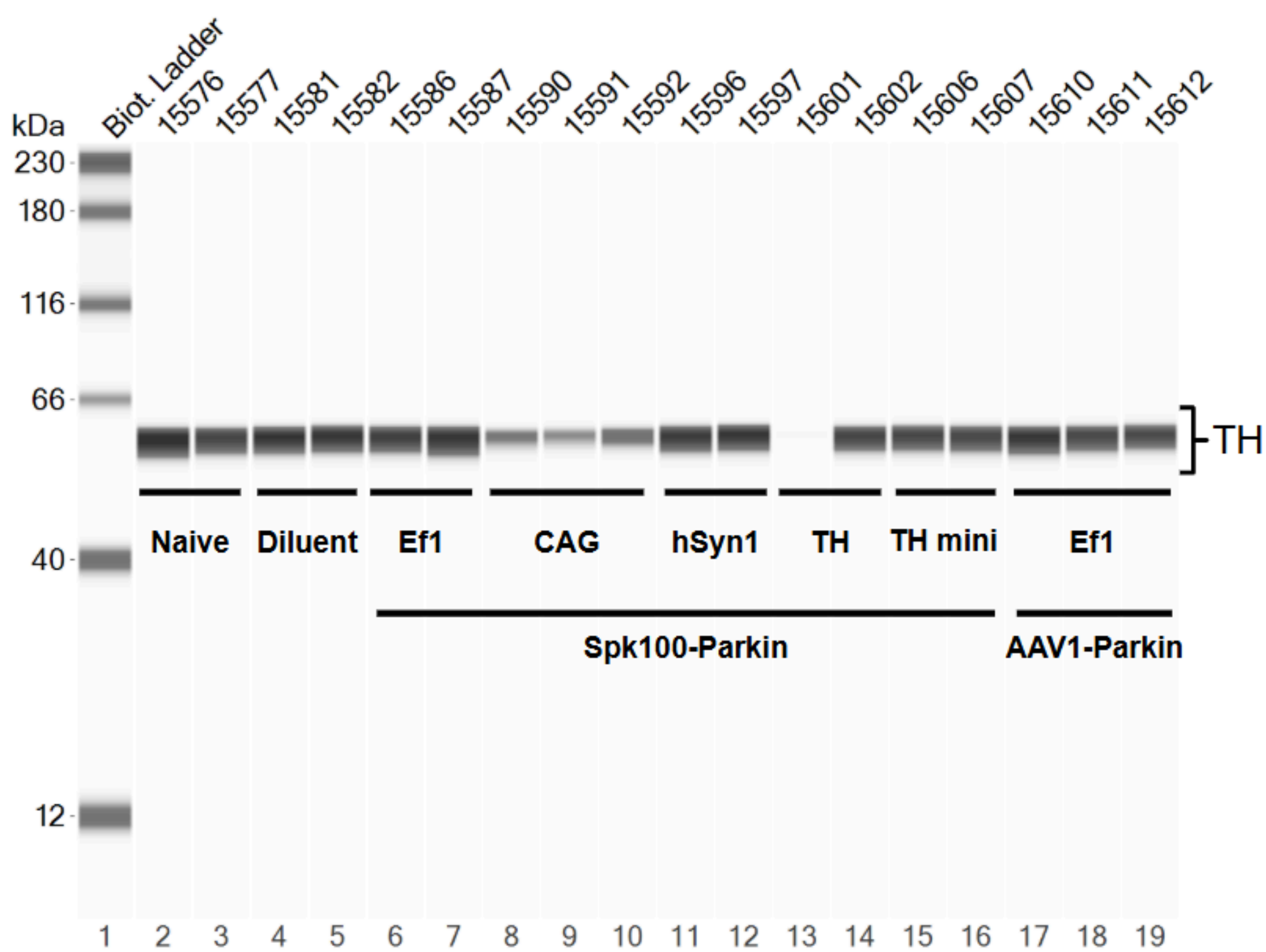

Biot. Ladder
15576
15577
15581
15582
15586
15587
15590
15591
15592
15596
15597
15601
15602
15606
15607
15610
15611
15612
kDa
230
180
116
66
40
12
TH
Naive
Diluent
Ef1
CAG
hSyn1
TH
TH mini
Ef1
Spk100-Parkin
AAV1-Parkin
1 2 3 4 5 6 7 8 9 10 11 12 13 14 15 16 17 18 19

(I)

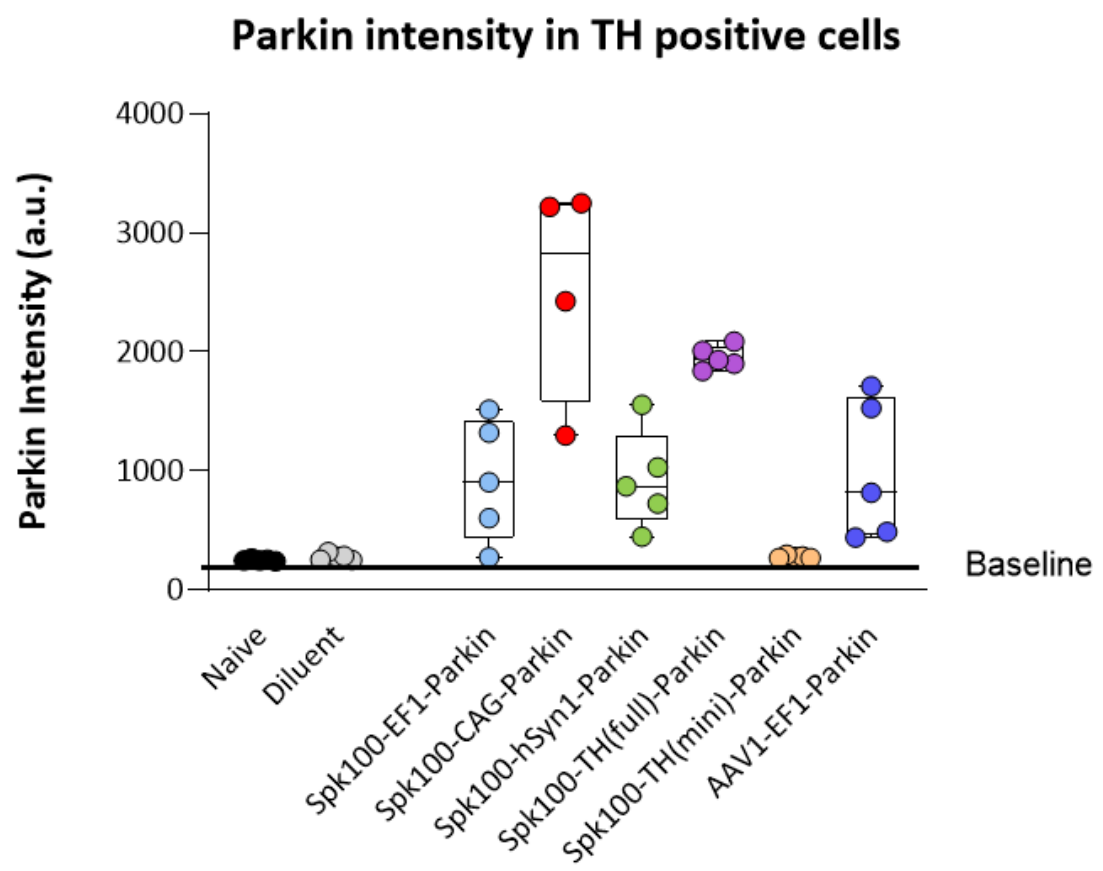

(J)

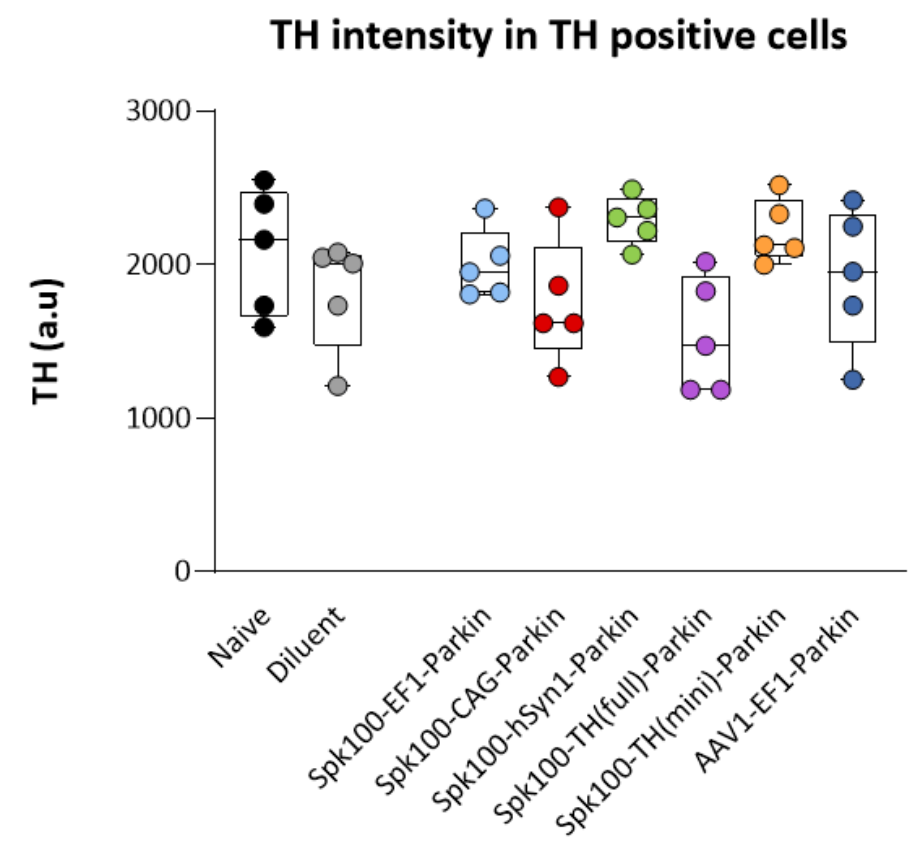

Capillary electrophoresis images showing human Parkin and TH expression of individual animals from SN (A-D) and striatum (E-H) following stereotaxic delivery of 1e10 vg/SN of Spark100-Ef1a-Parkin, Spark100-CAG-Parkin, Spark100-hSyn1-Parkin, Spark100-TH (full)-Parkin, or Spark100-TH (mini)-Parkin to the SN (I-J) Semi-Quantitative immunohistochemistry readouts on Parkin intensity in TH positive dopaminergic neurons and TH intensity in TH positive dopaminergic neurons**.**

Supplementary Figure 6: **Evaluation of gliosis markers Iba1 and Gfap in mouse SN Tissues following overexpression of Parkin (human) from different promoters.**

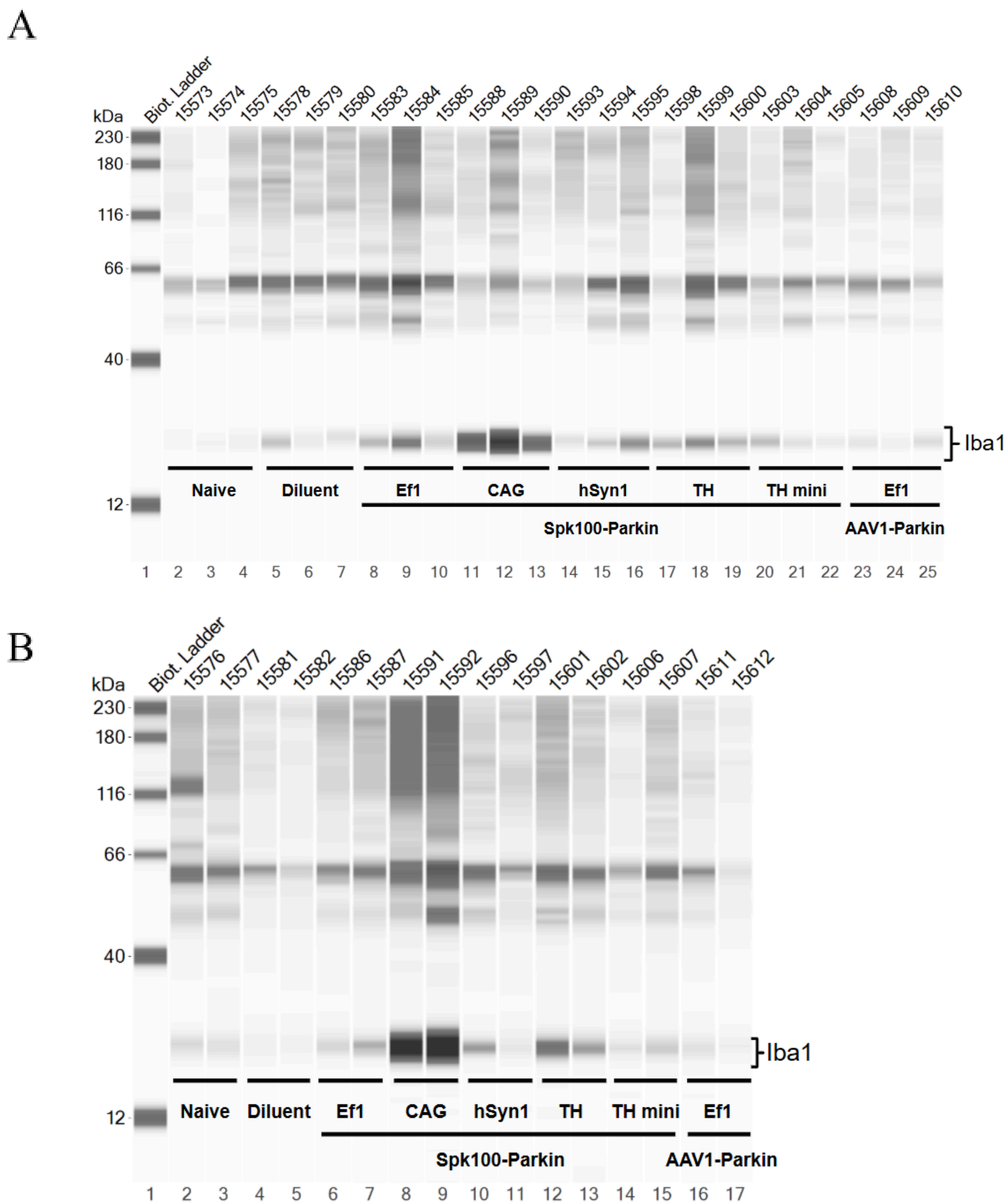

C

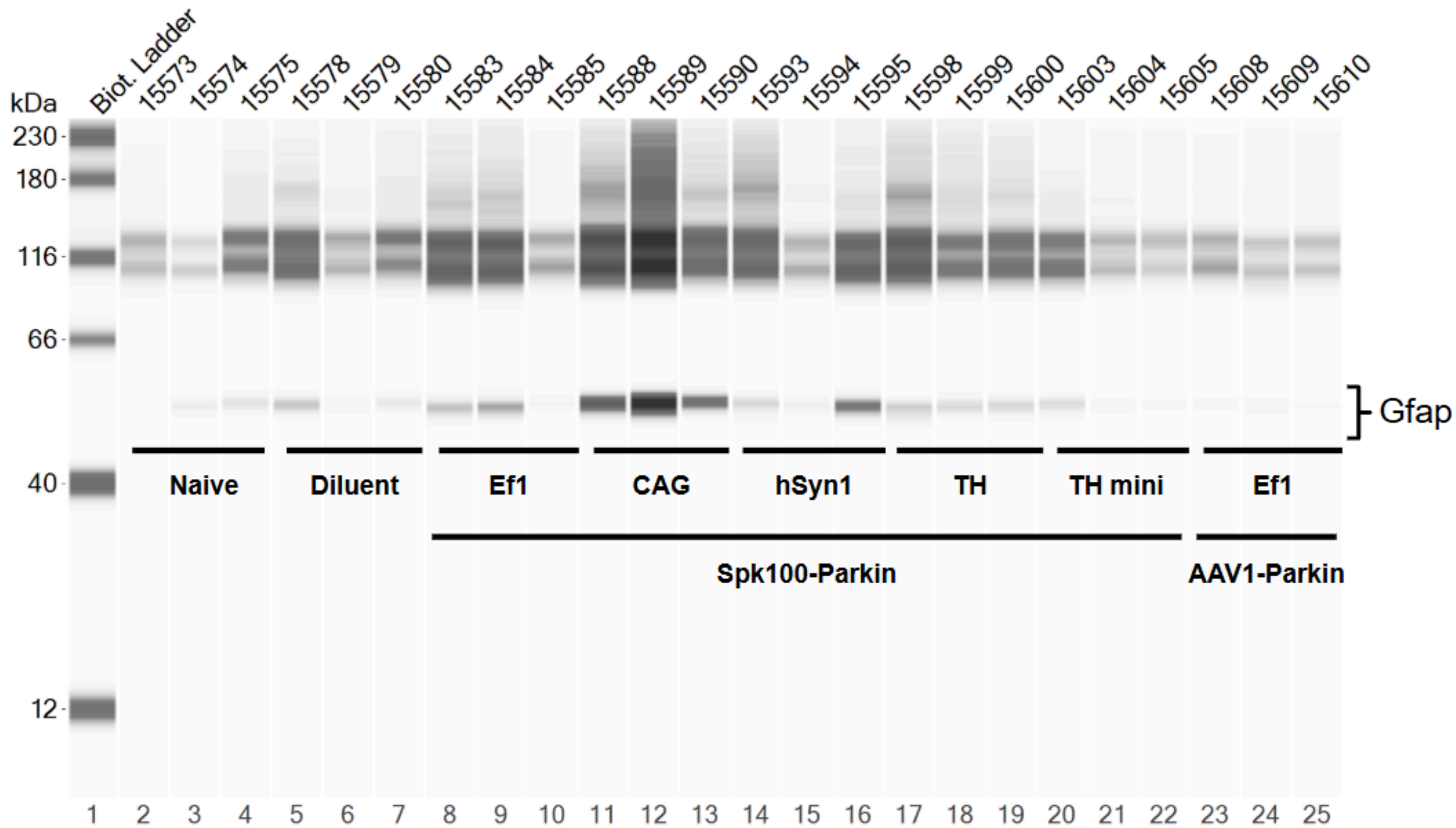

kDa
Biot. Ladder
15573
15574
15575
15578
15579
15580
15583
15584
15585
15588
15589
15590
15593
15594
15595
15598
15599
15600
15603
15604
15605
15608
15609
15610
230
180
116
66
40
12
Gfap
Naive
Diluent
Ef1
CAG
hSyn1
TH
TH mini
Ef1
Spk100-Parkin
AAV1-Parkin
1 2 3 4 5 6 7 8 9 10 11 12 13 14 15 16 17 18 19 20 21 22 23 24 25

D

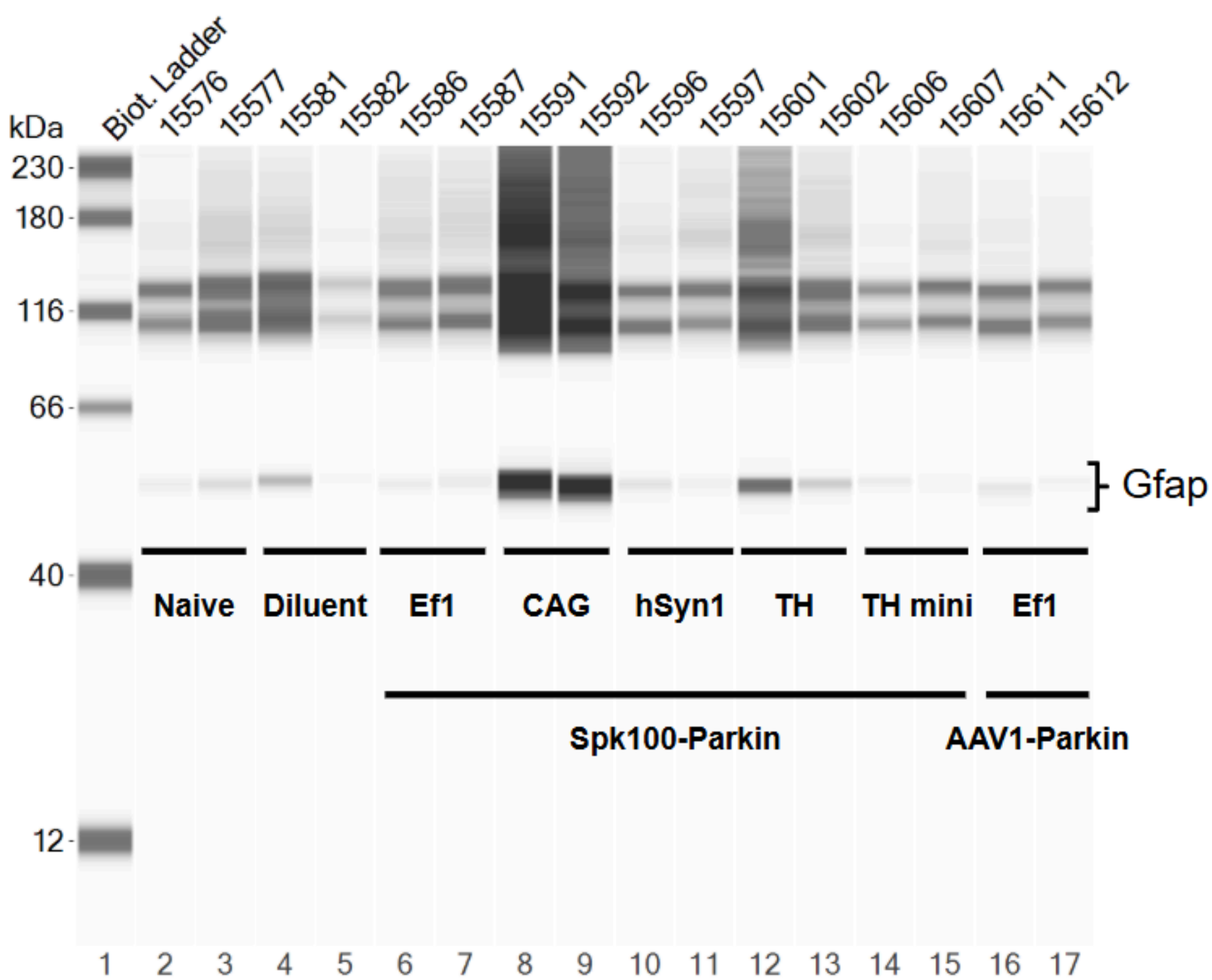

kDa
Biot. Ladder
15576
15577
15581
15582
15586
15587
15591
15592
15596
15597
15601
15602
15606
15607
15611
15612
230
180
116
66
40
12
Gfap
Naive
Diluent
Ef1
CAG
hSyn1
TH
TH mini
Ef1
Spk100-Parkin
AAV1-Parkin
1 2 3 4 5 6 7 8 9 10 11 12 13 14 15 16 17

Capillary electrophoresis images showing Iba1 (A,B) and Gfap (C,D) expression of individual animals from SN following stereotaxic delivery of 1.0×1010 vg/SN of Spark100-Ef1a-Parkin, Spark100-CAG-Parkin, Spark100-hSyn1-Parkin, Spark100-TH (full)-Parkin, or Spark100-TH (mini)-Parkin to the SN.
