# Supplementary material for: Restoring Parkin function: an AAV gene therapy approach for early-onset Parkinson’s disease
Source: Front Mol Neurosci. 2026 Oct 21;19:1939065. doi: 10.3389/fnmol.2026.1939065 (PMC13731301; doi:10.3389/fnmol.2026.1939065)
Supplement: Supplementary file 2 [file Data_Sheet_2.pdf]

Supplementary Materials and Methods

<u>In-vitro studies</u>

pUb^{Ser65} dot blot from SH-SY5Y cells:

Wild-type and Human Parkin knock out SH-SY5Y neuroblastoma cells were obtained from abcam (cat #ab280042). The control wild-type SH-SY5Y cells were provided with the Human Parkin knock out cells (cat #ab275475). SH-SY5Y cells were cultured and maintained as per manufacturer's instruction, with the exception of DMEM F-12 media (Gibco, 11320033) being used instead of EMEM.

WT and Parkin knock out SH-SY5Y cells were plated in 6 well plates at 1e6 cells per well and treated with 20uM FCCP (Cayman Chemical, Item# 15218) diluted in Dimethyl sulfoxide (DMSO; Sigma-Aldrich, Catalog #D2650) or vehicle (DMSO) for 1, 2, 4 and 6 hours. Post-treatment the cells were collected, and the extracted protein was quantified using standard BCA assay (Thermo Fisher, Cat. No. 23225). 2.5ug of protein was diluted in a total of 25ul using tris-buffered saline (TBS) and used for a dot-blot analysis of pUb^{Ser65}. The antibody details are included in the table below.

In brief, 1ug diluted protein (10uL) lysate was loaded onto a nitrocellulose membrane on a microfiltration blotting device with an attached vacuum manifold base (BioRad Cat #1706545). Total protein concentration was determined for all lysates, and a uniform amount of total protein was loaded per sample for the dot-blot assay. Samples were incubated on the membrane at RT for 1 hour. Blocking solution (LiCor Intercept Cat#927-80001) was added and incubated on an orbital shaker for 1 hour at RT. Primary antibody solution (1:500 Phospho-Ubiquitin Ser65 Cell Signaling Cat# 62802 diluted in 20mL 0.1% Tween blocking solution) was added and incubated

overnight with gentle rocking at 4C. The following day, 3 washes with TBST (TBS+0.1% Tween) were performed. Secondary antibody solution (Licor IR800 Cat# 926-32213 diluted 1:20,000 in blocking solution) was added and incubated with gentle rocking for 1 hour at RT. Membrane was scanned on LiCor Odyssey for blot quantitation.

SH-SY5Y localization of Parkin and mito-RFP upon FCCP treatment:

SH-SY5Y cells (WT and Parkin knock out) were plated on ready-to-use PDL coated 96-well plates (Perkin Elmer) at 20,000 cells/well on Day 0. Depending on the treatment paradigm on day 1 cells were either treated with Diluent or AAV1-Ef1a-Parkin (human) (stock titer 1.04e13vgs/ml) at multiplicity of infection (MOI) of 1e5vp (vector particles)/cell for 20,000 cells. On Day 2, mitochondria were tagged using mito-RFP (catalog #C10601 Invitrogen) at a MOI of 40 particles/cell. 24 hours after the labeling of mitochondria, cells were treated with 20uM of FCCP (stock concentration of 10mM) for 4hrs. After treatment with FCCP, cells were washed and fixed with 4% PFA for 15mins. This was followed by a standard immunocytochemistry protocol. Briefly, after the fixation step, cells were washed and permeabilized with PBS+0.5%TritonX100 for 15mins, washed, and blocked for 2hrs at RT with blocking buffer (PBS+0.1%Tween 20+10%Goat serum). Post blocking and washing, cells were incubated with Primary rabbit anti pUb^{Ser65} (Cell-signaling Cat #62802) at 1:250 or rabbit anti Parkin (Invitrogen Cat #702785) at 1:500 dilution overnight in Antibody buffer (PPBS+0.1%Tween 20+1%Goat serum) at 4C. The next day, primary antibody was aspirated and cells were washed with a washing buffer (PBS+0.1%Tween 20) 5 times, 5 minutes each wash and incubated with Goat anti Rabbit conjugated to Alexa Fluor 488 (GFP) for 1hr at RT. Cells were washed, treated with DAPI (1:1000) and imaged using Opera Phenix. Images were acquired using 10X air objective for EGFP, Cy3 and DAPI channel. The entire well image was

stitched together to qualitatively assess parkin (EGFP) colocalization to mitochondria (Cy3), resulting in yellow pixels post FCCP treatment.

Patient derived fibroblast ICC for pUb^{Ser65}:

Healthy control and Parkin-mutation (PRKN-PD) fibroblasts were obtained from NINDS. All participants gave written informed consent according to the Declaration of Helsinki to protocols approved by the Institutional Review Board of NINDS before undergoing research procedures. A detailed list of the specific mutations in the PRKN gene are listed in the results section. Patient fibroblast lines were established from 3-mm punch biopsies taken from the forearm. Cell lines were assayed at passage 11 or before. Cells were cultured in high glucose DMEM (Life Technologies, cat#11995073) with 10% fetal bovine serum.

For selection of optimal transduction conditions in fibroblasts, wild-type fibroblasts were seeded at 20000 cells/well in a 96-well plate format. Next day, the cells were transduced with 3 MOI of AAV-vectors (1e4, 1e5 or 1e6 vp/cell) expressing GFP from a CAG promoter (titers of each vector provided in the table below). 72hours post-transduction, the efficiency was assessed by imaging using Opera Phenix.

12 lines of primary patient-derived fibroblasts (except FPD075) were seeded in Ibidi slides (cat# 80827-90) at 20,000 cells per well. The next day cells were transduced with AAV2-CAG-Parkin (human) at an MOI of 1e4 vp/cell. After 72 hours of transduction, cells were treated with 1uM Valinomycin (Valinomycin, solid (SIGMA Cat#V0627-25MG) or an equivalent volume of vehicle (DMSO). After 3 hours of Valinomycin treatment fibroblasts were fixed and stained for pUb^{Ser65}following standard immunocytochemistry protocol. The antibodies used for this assessment are described in the table below. Slides were imaged using an Olympus FV3000 and

a 40X silicon oil immersion objective. Images were analyzed using ImageJ. For analysis, individual cells were outlined. An ROI of the mitochondrial region was created by converting the Cytochrome *c* slice into a binary mask. Measurements of the pUb^{Ser65} slice were taken within this mask. The mean grey value of pUb^{Ser65} was reported from individual cells. Fluorescence intensity data were normalized to the average of the baseline control (DMSO, no virus) values for each individual participant, with the mean of this baseline condition set to 1.

Table 1: Table showing a list of Healthy controls and PRKN-PD patient fibroblast lines, with specific Parkin mutations (s), disease status and age of onset of disease.

Sample Name	PD	Mutation	PD AAO	Sex
FPD014	No	Does not have biallelic mutations in Parkin	N/A	M
FPD038	No	Does not have biallelic mutations in Parkin	N/A	M
FPD069	No	Does not have biallelic mutations in Parkin	N/A	M
FPD070	No	Does not have biallelic mutations in Parkin	N/A	M
FPD009	Yes	Parkin homo c.1244C>A; p.Thr415Asn	27	F
FPD018	Yes	Parkin compound het (Exon11 del; p.G429D)	31	M
FPD019	Yes	Parkin compound het (Exon11 del; p.G429D)	37	F
FPD036	Yes	Parkin compound het (c.167 T>A, p.Val56Glu; Partial gene deletion including E7	35	F
FPD037	Yes	Parkin compound het (c.167 T>A, p.Val56Glu; Partial gene deletion including E7	32	F
FPD061	Yes	Parkin compound het (p.R275W, del E3-4)	19	M
FPD062	Yes	Parkin compound het (p.R275W, del E3-4)	37	M

FPD068	Yes	Parkin compound het VUS splice site (rs1204087943) and E3-4 deletion	27	F
FPD075	Yes	Parkin compound het E3 del and E3-E4 del	37	M

Vector Production

Recombinant AAV vectors were produced at the Research Vector Core at Spark Therapeutics Inc. by transfecting adherent HEK293 cells using a calcium-phosphate-based triple plasmid system containing pHelper, pRepCap, and a transgene plasmid (for Parkin) at a 1:1:1 mass ratio. After 96 hours, the cells were harvested and lysed by sonication to release the viral particles. The lysate was then treated with benzonase to digest unwanted host cell DNA and RNA, and the AAVs were concentrated using polyethylene glycol (PEG) precipitation. For final purification, the vectors were isolated via CsCl density-gradient ultracentrifugation and then buffer-exchanged into a PBS/Pluronic F-68 solution through dialysis. The quality of the final product was thoroughly checked: vector genomes were quantified using TaqMan qPCR with BGH polyA-specific primers (forward 5′-CTTGCCTTCCTTGACCCT-3′, reverse 5′-CCCAGAATAGAATGACACCTACT-3′) and a probe (56-FAM/TTAGGAAAG/ZEN/GACAGTGGGAGTGGC/3IABkFQ); capsid protein purity was confirmed by SDS-PAGE with a Sypro Ruby stain; and endotoxin levels were verified to be less than 1 EU/mL. The final, quality-controlled vectors were stored at −80°C until they were needed [16].

Short-term Tolerability/ Dose Ranging Study

The study included 24 male CD® Sprague Dawley rats (n=4/5 per group) aged 8 to 10 weeks at dosing (Day 0). Animals remained untreated (Group 1, n=4), received the diluent (Group 2, n=5) or the AAV1-Ef1a-Parkin (human), stock titer 1.04e13vgs/ml at dose 3.0×10^{10} (Group 5, n=5), 3.0×10^{9} (Group 4), or 3.0×10^{8} vg/SN (Group 3, n=5), and were monitored until necropsy 8 weeks post-dosing. The animals were randomized during surgical procedures to avoid any surgical bias. The rat tolerability study was conducted at Charles River Laboratories, Finland and was performed as specified in the license authorized by the national Animal Experiment Board of Finland and according to the National Institutes of Health (Bethesda, MD, USA) guidelines for the care and use of laboratory animals.

Twenty-four animals received bilateral intraparenchymal injections into the SNpc at the following coordinates: AP: 5.2 (posterior from bregma), ML: +/- 2.2, and DV: -7.5 (from the brain surface). AAV1-Ef1a-Parkin (human) were injected at a volume of 3uL/hemisphere using 10uL Hamilton syringe with a 26 G beveled needle. Animals were maintained under 2.5% isoflurane anesthesia for the length of the surgery. After surgery the skin was closed and disinfected. Animals were carefully monitored by lab personnel twice a day following surgery.

Rats were then transcardially perfused with ice cold heparinized (Heparin 2.5 IU/ml) saline to remove blood from the brains. The brains were removed carefully and were freshly dissected to be used for biochemical analysis (flash frozen, described in the section sample processing) or Immunohistochemistry (Fixed, described in the section immunostaining).

Promoter assessment

In-house mouse studies were approved by the Institutional Animal Care and Use Committee (IACUC) of Rowan University, Protocol No. 2020-1215 [2018-007]. Forty male animals (Mus

musculus / C57BL/6) from Jackson Laboratories (Bar Harbor, ME, stock number# 000664) aged between 8-12 weeks received bilateral intraparenchymal (IPa) injections into the SNpc at the following coordinates: AP -3.1, ML ± 1.2, and DV -4.3, from the top of the skull and relative to the Bregma. The test articles (listed below in the AAV-vectors section) were injected at a volume of 2 μL per brain hemisphere and at a 0.4 μL/min rate. Animals were maintained under gas anesthesia using 3% isoflurane (via nose cone, at ~1 L/min) throughout the procedure. Neosporin was applied after the stitches were placed to close the wound, and mice received carprofen (5 mg/kg subcutaneously). Animals were monitored by the Rowan University animal care staff on Days 1 and 2 post-surgery. Additional carprofen was given if needed.

On study Day 0, animals remained untreated (Group 1), received the diluent (Groups 2), or the following test articles at 1.0×10^{10} vg/SN: Spark100-Ef1a-Parkin (human) (Group 3), Spark100-CAG-Parkin (human) (Group 4), Spark100-hSyn1-Parkin (human) (Groups 5), Spark100-Th(full)-Parkin (human) (Groups 6), Spark100-TH(mini)-Parkin (human) (Groups 7) and AAV1-Ef1a-Parkin (human) (Groups 8). All animals received bilateral intraparenchymal (IP) injections into the SNpc and were monitored for up to 2 months (56 days) after injection.

On study Day 56, animals were sacrificed by live decapitation using a guillotine. The brains were removed carefully and were freshly dissected to be used for biochemical analysis (flash frozen, described in the section sample processing) or Immunohistochemistry (Fixed, described in the section immunostaining).

The sequence of the promoters is listed in the Supplementary files.

Table 2: List of vectors used for in-vitro Studies

Vector name	Titer (vg/ml)
AAV1-CAG-GFP	4.72×10^{12}
AAV2-CAG-GFP	1.19×10^{13}
AAV2retro-CAG-GFP	3.67×10^{12}
AAV5-CAG-GFP	2.78×10^{13}
AAV6-CAG-GFP	4.35×10^{12}
AAV8-CAG-GFP	3.63×10^{13}
AAV9-CAG-GFP	2.37×10^{13}
Spk100-CAG-GFP	1.39×10^{13}
Spk200-CAG-GFP	2.09×10^{13}
AAV1-Ef1a-Parkin (human)	1.04×10^{13}
AAV2-CAG-Parkin (human)	2.43×10^{13}

Table 3: List of vectors used for in-vivo Studies

Name	Vector Titer (vg/mL)
Spark100-Ef1a- Parkin (human)	3.56×10^{13}
Spark100-CAG- Parkin (human)	1.14×10^{13}
Spark100-hSYN1- Parkin (human)	7.92×10^{13}
Spark100-TH (full)- Parkin (human)	3.01×10^{13}
Spark100-TH (mini)- Parkin (human)	1.71×10^{13}
AAV1-Ef1a-Parkin (human)	1.04×10^{13}

Protein Assays

Sample Processing

Dissected right SN samples were suspended in 500 uL radioimmunoprecipitation assay (RIPA) buffer (Thermo Fisher, Cat. No. PI89901) +PIC (Thermo Fisher, Cat. No. 78440) and homogenized using the TissueLyser II (QIAGEN, Cat. No. 85300) at 25.0 Hz for 30 seconds twice, switching sample arms between the repeats.

Total protein concentration in homogenized samples was determined with the BCA method using a commercial kit (Thermo Fisher, Cat. No. 23225) performed according to manufacturer's instructions.

Capillary Electrophoresis

Capillary electrophoresis was utilized in place of conventional Western blotting due to its requirement for significantly lower protein input—which is critical for micro-dissected substantia nigra tissues—and its highly reproducible, automated quantification.

Protein lysate was diluted 1:5 in the provided sample buffer, then mixed with the master mix and heated at 75˚C for 10 min. Protein mix containing 1 µg of protein, protein normalization reagent, blocking reagent, wash buffer, biotinylated ladder, primary antibodies (Table 4), and chemiluminescent substrate were dispensed into designated wells in a manufacturer-provided microplate. The plate was loaded into the instrument (Protein Simple, Cat #004-650.), and proteins were drawn into individual capillaries on a 25 capillary cassette (12–230 kDa). Data was analyzed using Compass software (v6.0 or higher) provided by the manufacturer. Protein concentrations were normalized to the total protein content in the capillary and reported as "corrected area under the curve" (A.U.C.). Values under 10,000 A.U.C. were considered below the level of quantitation (BLOQ).

pUb^{Ser65} ELISA

Assay was performed using the Cell Signaling PathScan pUb^{Ser65} Sandwich ELISA Kit (Cat#28384). An amount of protein lysate totaling 5ug of was added into Sample Diluent to a final volume of 100uL and loaded onto the plate. The plate was sealed and incubated at 37C for

2 hours with light shaking (250 RPM). Plate was washed 4 times with 200uL 1X wash buffer and wells were aspirated after the final wash. 100uL reconstituted antibody was added and the plate was again sealed and incubated at 37C for 1 hour. Previous wash steps and aspiration of wells were repeated. 100uL reconstituted HRP-linked secondary antibody was added, plate was sealed and incubated at 37 for 30 minutes. Wash step with aspiration was again repeated. 100uL of TMB Substrate was added and the plate was incubated in the dark at RT for 30 minutes. A 100uL stop solution was then added and plate was read on Biotek Synergy H1 Multimode Reader with Agilent Gen5 software. Absorbance was read at 450nm, and the standard curve was fit using a four-parameter logistic curve. Sample inputs were standardized by total protein concentration, and pUbSer65 levels were quantified utilizing the kit's internal standard.

Immunostaining

Forty formaldehyde-perfused and fixed mouse brain hemispheres (one hemisphere per brain) were cryoprotected with 0.1 M phosphate buffer (PB, pH 7.4) containing 20% sucrose for 72 hours, and then rapidly frozen. Serial cryostat sections (30-µm) were cut coronally through the brainstem containing the substantia nigra (SN), approximately from Bregma -2.46 mm to -3.88 mm.

One set of sections was processed for double-immunofluorescence labeling (TH + Parkin). Following washes in 0.01 M phosphate buffered saline (PBS, pH 7.4), sections were incubated free-floating in PBS containing 0.3% Triton X-100, 2% blocking serum and 2 primary antibodies for 43 hours at 4°C. This was followed by incubation of sections in PBS containing 2 separate secondary antibodies conjugated with Alexa Fluor® 594 (for TH) and Alexa Fluor® 488 (for Parkin), respectively. Sections were then counterstained with FD DAPI solution™ (FD Neurotechnologies, Columbia, MD). All above steps were carried out at room temperature

except where indicated, and each step was followed by washing in PBS. After thorough washes in PBS, all sections were mounted on gelatin-coated slides (4 sections per slide), cover slipped with Vectashield® (Vector Lab, Burlingame, CA), sealed with nail polish, and stored at 4ºC.

A second set of sections was processed for triple-immunofluorescence labeling (TH + Iba1 + GFAP). All methods are identical to the above description with the exception of the secondary antibody incubation, which was performed in PBS containing 3 separate secondary antibodies conjugated with Alexa Fluor® 647 (for TH), Alexa Fluor® 594 (for Iba1) and Alexa Fluor® 488 (for GFAP).

Twenty-four PFA-perfused and fixed rat brain hemispheres (one hemisphere per brain) were cryoprotected with 0.1 M phosphate buffer (PB, pH 7.4) containing 20% sucrose for 72 hours, and then rapidly frozen. Serial cryostat sections (30-µm) were cut coronally containing the substantia nigra (SN). One set of sections was processed for double-immunofluorescence labeling (TH + Parkin following standard IHC procedure). After initial washes, sections were blocked with 5% BSA and 0.3% Triton X100 for 30 minutes and then incubated with primary antibody (please refer to the antibody list below) overnight at 4C. Sections were then incubated with a secondary antibody for 1.5 hours at room temperature. Washes with PBS were performed in between each step.

All imaging was performed on a Zeiss Axioscan Z1 using a 5x air objective at 0.91 µm per pixel.

HPLC Analysis of Catecholamines and Acidic Metabolites

Dopamine (DA), 3,4-dihydroxyphenylacetic acid (DOPAC), homovanillic acid (HVA) and serotonin (5-HT) concentrations in right striatal tissue samples were determined by high performance liquid chromatography (HPLC) with electrochemical detection.

The frozen tissue samples were homogenized (1:10 w/v) in a 0.1 M acetic acid, 1 mM oxalic acid, 3 mM L-cysteine - 0.2 M perchloric acid mixture (1:6) with ultrasonic processor (SONOPULS mini20 with MS 1.5 microtip, Bandelin Electronic GmbH & Co, Berlin, Germany). Tissue homogenates were centrifuged for 10 min at 17000 g at 4 °C. Supernatants were collected, centrifuged again, and transferred into glass vials for analysis.

The Infinity II 1260 HPLC system (Agilent Inc., Santa Clara, CA, USA) consists of a G711A solvent delivery system, G7129A thermostatic autosampler, amperometric electrochemical detector (Logichrom, Lablogic Systems Ltd., Sheffield, UK) equipped with Sencell GC 2mm flow cell, and a salt-bridge reference electrode. The HPLC system is controlled by the LAURA control and analysis software (LabLogic Systems Ltd.). The analytes were detected with a 0.78 V working electrode potential.

The analytes were separated on a Kinetex C-18 reversed-phase column, 4.6 ´ 100 mm, 2.6 µm, with a SecurityGuard Ultra precolumn for C18 columns (Phenomenex ApS, Værløse, Denmark) in an isocratic run. The column and electrochemical detector were maintained at 55 °C. The mobile phase is 100 mM phosphoric acid, 100 mM citric acid, 0.6 g/L 1-octanesulfonic acid, 12 mM NaCl and 0.1 mM disodium EDTA, pH 3.05 – acetonitrile mixture (1000:85, v/v). The flow rate was set to 1 mL/min and injection volume to 10 µL. The levels of the analytes were calculated using external standards and expressed as pg/g wet tissue.

Table 7. List of Primary Antibodies used in the study

Antigen		Host Species	Target Species	Tag	Dilution	Manufacturer	Cat. No.	Assay
Primary Antibodies	GFAP	Rat	H, Ms, Rat	Unconjugated	1:250	ThermoFisher	13-0300	IHC
	IBA1	Rabbit	H, Ms, Rat	Unconjugated	1:500	FUJIFILM	019-19741	IHC
	TH	Sheep	Mammalian	Unconjugated	1:500	Pel-Freez	P60101-150	IHC
	Parkin	Rabbit	H, Ms, Rat	Unconjugated	1:1000	Thermo Fisher	702785	IHC
	GFAP	Rabbit	H, Ms, Rat	Unconjugated	1:100	Cell Signaling	12389	JESS
	IBA1	Rabbit	H, Ms, Rat	Unconjugated	1:50	Abcam	ab178847	JESS
	TH	Rabbit	Mammalia	Unconjugated	1:100	Pel-Freez	P40101-150	JESS
	Parkin	Mouse	H, Ms, Rat	Unconjugated	1:100	Cell Signaling	4211	JESS
	Parkin	Rabbit	H, Ms, Rat	Unconjugated	1:100	Thermo Fisher	702785	JESS
	Parkin	Mouse	H, Ms, Rat	Unconjugated	1:250	Santa-Cruz	sc-32282	ICC fibroblast
	pUbSer65	Rabbit	H	Unconjugated	1:250	Cell Signaling	62802	ICC fibroblasts
	pUbSer65	Rabbit	H	Unconjugated	1:500	Cell Signaling	62802	Dot Blot

GFAP = glial fibrillary acidic protein; H = human; IBA1 = ionized calcium-binding adapter molecule 1; Ms = mouse; TH = tyrosine hydroxylase; Uncomj. = unconjugated.

Statistical analysis

All quantitative data in the figures, unless otherwise specified, are presented as mean ± SD and analyzed using GraphPad Prism software (version 10.3.0) or MS Excel. Comparisons between the two groups were done using unpaired two-tailed Student's t test. Differences between more than two groups were carried out using 1-way ANOVA with Tukey's or Dunnett's correction for relevant pairwise comparisons. For all statistical analysis, $p < 0.05$ was considered significant. The statistical analysis performed for each dataset is indicated in the figure legends.

Sequence of Promoters

CAG:

ATCTAGTTATTAATAGTAATCAATTACGGGGTCATTAGTTCATAGCCCATATATGGAGTTCCGCGTTACATAACTTACGGTAAATGGCCC
GCCTGGCTGACCGCCCAACGACCCCCGCCCATTGACGTCAATAATGACGTATGTTCCCATAGTAACGCCAATAGGGACTTTCCATTG
ACGTCAATGGGTGGAGTATTTACGGTAAACTGCCCACTTGGCAGTACATCAAGTGTATCATATGCCAAGTACGCCCCCTATTGACGTC
AATGACGGTAAATGGCCCGCCTGGCATTATGCCCAGTACATGACCTTATGGGACTTTCCTACTTGGCAGTACATCTACGTATTAGTCAT
CGCTATTACCATGGTCGAGGTGAGCCCCACGTTCTGCTTCACTCTCCCCATCTCCCCCCCCTCCCCACCCCCAATTTTGTATTTATTTA
TTTTTTAATTATTTTGTGCAGCGATGGGGGCGGGGGGGGGGGGGGGGCGCGCGCCAGGCGGGGCGGGGCGGGGCGAGGGGCGGG
GCGGGGCGAGGCGGAGAGGTGCGGCGGCAGCCAATCAGAGCGGCGCGCTCCGAAAGTTTCCTTTTATGGCGAGGCGGCGGCGGC
GGCGGCCCTATAAAAAGCGAAGCGCGCGGCGGGCGGGAGTCGCTGCGCGCTGCCTTCGCCCCGTGCCCCGCTCCGCCGCCGCCTC
GCGCCGCCCGCCCCGGCTCTGACTGACCGCGTTACTCCCACAGGTGAGCGGGCGGGACGGCCCTTCTCCTCCGGGCTGTAATTAGC
GCTTGGTTTAATGACGGCTTGTTTCTTTTCTGTGGCTGCGTGAAAGCCTTGAGGGGCTCCGGGAGGGCCCTTTGTGCGGGGGGAGC
GGCTCGGGGGGTGCGTGCGTGTGTGTGTGCGTGGGGAGCGCCGCGTGCGGCTCCGCGCTGCCCGGCGGCTGTGAGCGCTGCGGGC
GCGGCGCGGGGCTTTGTGCGCTCCGCAGTGTGCGCGAGGGGAGCGCGGCCGGGGGCGGTGCCCCGCGGTGCGGGGGGGGCTGCG
AGGGGAACAAAGGCTGCGTGCGGGGTGTGTGCGTGGGGGGGTGAGCAGGGGGTGTGGGCGCGTCGGTCGGGCTGCAACCCCCCC
TGCACCCCCCTCCCCGAGTTGCTGAGCACGGCCCGGCTTCGGGTGCGGGGCTCCGTACGGGGCGTGGCGCGGGGCTCGCCGTGCC
GGGCGGGGGGTGGCGGCAGGTGGGGGTGCCGGGCGGGGCGGGGCCGCCTCGGGCCGGGGAGGGCTCGGGGGAGGGGCGCGGCG
GCCCCCGGAGCGCCGGCGGCTGTCGAGGCGCGGCGAGCCGCAGCCATTGCCTTTTATGGTAATCGTGCGAGAGGGCGCAGGGACT
TCCTTTGTCCCAAATCTGTGCGGAGCCGAAATCTGGGAGGCGCCGCCGCACCCCCTCTAGCGGGCGCGGGGCGAAGCGGTGCGGC
GCCGGCAGGAAGGAAATGGGCGGGGAGGGCCTTCGTGCGTCGCCGCGCCGCCGTCCCCTTCTCCCTCTCCAGCCTCGGGGCTGTC
CGCGGGGGGACGGCTGCCTTCGGGGGGGACGGGGCAGGGCGGGGTTCGGCTTCTGGCGTGTGACCGGCGGCTCTAGAGCCTCTGC
TAACCATGTTCATGCCTTCTTCTTTTTCCTACAGCTCCTGGGCAACGTGCTGGTTATTGTGCTGTCTCATCATTTTGGCAAAGAATT

EF1a:

GGGCAGAGCGCACATCGCCCACAGTCCCCGAGAAGTTGGGGGGAGGGGTCGGCAATTGAACCGGTGCCTAGAGAAGGTGGCGCG
GGGTAAACTGGGAAAGTGATGTCGTGTACTGGCTCCGCCTTTTTCCCGAGGGTGGGGGAGAACCGTATATAAGTGCAGTAGTCGCC
GTGAACGTTCTTTTTCGCAACGGGTTTGCCGCCAGAACACAG

hSYN11:

AGTGCAAGTGGGTTTTAGGACCAGGATGAGGCGGGGTGGGGGTGCCTACCTGACGACCGACCCCGACCCACTGGACAAGCACCCA
ACCCCCATTCCCCAAATTGCGCATCCCCTATCAGAGAGGGGGAGGGGAAACAGGATGCGGCGAGGCGCGTGCGCACTGCCAGCTT
CAGCACCGCGGACAGTGCCTTCGCCCCCGCCTGGCGGCGCGCGCCACCGCCGCCTCAGCACTGAAGGCGCGCTGACGTCACTCGC
CGGTCCCCCGCAAACTCCCCTTCCCGGCCACCTTGGTCGCGTCCGCGCCGCCGCCGGCCCAGCCGGACCGCACCACGCGAGGCGC

GAGATAGGGGGGCACGGGCGCGACCATCTGCGCTGCGGCGCCGGCGACTCAGCGCTGCCTCAGTCTGCGGTGGGCAGCGGAGGA

GTCGTGTCGTGCCTGAGAGCGCAG

TH (Full):

CTGCCTCCAGAGAGCCTGGCCCCAAGGAAGAGTCTAGTAAGCTTAGTTCCCATCGGGCTTCCATGAAAGCACAACTGGCCCGGCAG

GAAACCGAATTAAAAAGCAATATTTGTATCAGTGGAAGACATTTGCTGAAAGGTTAAATCCACATCCGGCAGTGTGGGCCATGAGCC

TCCGGCGTGGTGTTCATCAGGCATGTCTCTCCTCCTGGCCTGGGCACCTGAGCACTGGGGCTGCCCTGGGCAGAGCTGGGGCAGGG

TGCTGGGGGGCCTGGAGCTGCCTCACCGAGGGATCCTCAGCAGCCGACCCTGGGGGAGGCAAATGAGACTCTTTCTGGGGACCTT

GAGGGGAGCTCGGGGGAGCCATGCAGAGCTTCACCAGGCCTGGACACTGGGCATGGAGGCTGGGCCACCCAAGGGCCATCACCA

GGGACTCAGGTGGGTGGGCCTCAGCCCTGGGTGACAGAAGCTCACGGGCTGCAGGGCGAGGCCAGAGGCTGAGCCTTCAGGCTG

AGGTCTTGGAGGCAAATCCCTCCAACGCCCTTCTGAGCAGGCACCCAGACCTACTGTGGGCAGGACCCACAGGAGGTGGAGGCCT

TTGGGGAACACCGTGGAGGGGCATAGCATCTCCGAGAGAGGACAGGGTCTGCACTGGGTGCTGAGAGACAGCAGGGGCCGAGCG

GTAGGCTTCCCTGCCCCCAGGGATGTTCCAGGGGAGCGCAAGGGAGGGGCATTAATATCGTGGCAAGAAAGGGCAGGCATTGCAG

AGTGAGCAGCGACGGAACTGGGTTTTGTGGGATGCATAGGAGTTCACCCGGATAAGAGGTGGGTGAGGAATGACACTGCAAACCG

GGGATCACGGAGCCCCAAATCCTTCTGGGCCAGGAAGTGGGAAGGGTTGGGGGGTCTTCCCTTTGCTTTGACTGAGCACTCAGCCT

GCCTGCAGAGGGCAGCGAGGAGCCACGGAGGGGTGTGGGACAGGGATGCCATGGCTGAAGCAGTTTTAGGAAAGGTCCCAGGGG

CTATTGTTGAAGAGAGAACGGGGAGCGGGGAGTCCCACAGCTGACAGGAGCAGAGTGGGCCCTGAGAGATGCCAGCTCTGGGTG

CCACAGTGACCAGCCGGGGTAGGCCTTCGAGAAGTCAGGGAGCGTCTAGGGCTTCTGGCTCCTGCTGGGCCCAGGGTGTCATCTTG

GGCTGCCAACACCAGAAAGCCCAGCAGATACAGGAAGCCCCAAGCCCTGTCGGAAACGGTTCTTCTCCAGGAGGGACAGCGGTG

GCAGCGTTCAGCCGCAGGCCATGCACTCTGGGGCCACGTCCTTCCCTCTGTACAGTCCAGCATTGTCAAGGCGGGCTCTGGCCATC

TCTGCTGACCCCAGAGGGATGGGGAGGCCTCCCCTTCCACCAGAAGGGCCAGAAGCCACCCTGGGCAGGGGCATCACTCTCCCTG

GGTGGGGCAGCGGCGGGGAGCAGGAGGTGCCAGTGGGCGTGGGCTGGATGCGGGTGCCTGCGGGGCGGACATGGAACTTGGGGG

AGGCTCTAGGCTGGGGTTGTCCTCAAGGGAGTTCTCAGGTCACCCCAGGGTCACCCTCAACCCGGGGCCTGGTGGGGTAGAGGAG

AAACTGCAAAGGTCTCTCCAAGGGGAAGGCATCAGGGCCCTCAGCACTGAGGGACGTGCGTGCTCTTCAAAGAAGGGGCCACAG

GACCCCGAGGGAAGCCAGGAGCTAGCAGTGGGCCATAGAGGGGCTGAGTGGGGTGGGTGGAAGCCGTCCCTGGCCCTGGTCGCC

CTGGCAACCCTGGTGGGGACTGTGATGCAGGAGGTGGCAGCCATTTGGAAACGCGTGGCGTCTCCTTAGAGATGTCTTCTTCAGCC

TCCCAGGGTCCTCCACACTGGACAGGTGGGCCCTCCTGGGACATTCTGGACCCCACAGGGCGAGCTTGGGAAGCCGCTGCAAGGG

CCACACCTGCAGGGCCCGGGGGCTGTGGGCAGATGGCACTCCTAGGAACCACGTCTACAAGACACACGGCCTGGAATCTTCTGGA

GAAGCAAACAAATTGCCTCCTGACATCTGAGGCTGGAGGCTGGATTCCCCGTCTTGGGGCTTTCTGGGTCGGTCTGCCACGAGGTT

CTGGTGTTCATTAAAAGTGTGCCCCTGGGCTGCCAGAAAGCCCCTCCCTGTGTGCTCTCTTGAGGGCTGTGGGGCCAAGGGGACCC

TGGCTGTCTCAGCCCCCCGCAGAGCACGAGCCCCTGGTCCCCGCAAGCCCGCGGGCTGAGGATGATTCAGACAGGGCTGGGGAGT

GAAGGCAATTAGATTCCACGGACGAGCCCTTTCTCCTGCGCCTCCCTCCTTCCTCACCCACCCCCGCCTCCATCAGGCACAGCAGGC

AGGGGTGGGGGATGTAAGGAGGGGAAGGTGGGGGACCCAGAGGGGGCTTTGACGTCAGCTCAGCTTATAAGAGGCTGCTGGGCC

AGGGCTGTGGAGACGGAGCCCGGACCTCCACACTGAGCCA

TH (mini):

AGACACACGGCCTGGAATCTTCTGGAGAAGCAAACAAATTGCCTCCTGACATCTGAGGCTGGAGGCTGGATTCCCCGTCTTGGGGC

TTTCTGGGTCGGTCTGCCACGAGGTTCTGGTGTTCATTAAAAGTGTGCCCCTGGGCTGCCAGAAAGCCCCTCCCTGTGTGCTCTCTT

GAGGGCTGTGGGGCCAAGGGGACCCTGGCTGTCTCAGCCCCCCGCAGAGCACGAGCCCCTGGTCCCCGCAAGCCCGCGGGCTGA

GGATGATTCAGACAGGGCTGGGGAGTGAAGGCAATTAGATTCCACGGACGAGCCCTTTCTCCTGCGCCTCCCTCCTTCCTCACCCA

CCCCCGCCTCCATCAGGCACAGCAGGCAGGGGTGGGGGATGTAAGGAGGGGAAGGTGGGGGACCCAGAGGGGGCTTTGACGTCA

GCTCAGCTTATAAGAGGCTGCTGGGCCAGGGCTGTGGAGACGGAGCCCGGACCTCCACACTGAGCCATGCCCACCCCCGACGCCA

CCACGCCACAGG
